# Unique Microbial Catabolic Pathway for the Human Core *N*-Glycan Constituent Fucosyl-α-1,6-*N*-Acetylglucosamine-Asparagine

**DOI:** 10.1128/mBio.02804-19

**Published:** 2020-01-14

**Authors:** Jimmy E. Becerra, Jesús Rodríguez-Díaz, Roberto Gozalbo-Rovira, Martina Palomino-Schätzlein, Manuel Zúñiga, Vicente Monedero, María J. Yebra

**Affiliations:** a Laboratorio de Bacterias Lácticas y Probióticos, Departamento de Biotecnología de Alimentos, IATA-CSIC, Paterna, Spain; b Departamento de Microbiología, Facultad de Medicina, Universidad de Valencia, Valencia, Spain; c NMR Facility, Centro de Investigación Príncipe Felipe, Valencia, Spain; University of Queensland

**Keywords:** *Lactobacillus casei*, alpha-l-fucosidase, core fucosylation, fucosylated *N*-glycopeptides, glycosylasparaginase

## Abstract

The survival of commensal bacteria in the human gut partially depends on their ability to metabolize host-derived molecules. The use of the glycosidic moiety of *N*-glycoproteins by bacteria has been reported, but the role of *N*-glycopeptides or glycoamino acids as the substrates for bacterial growth has not been evaluated. We have identified in Lactobacillus casei strain BL23 a gene cluster (*alf-2*) involved in the catabolism of the glycoamino acid fucosyl-α-1,6-*N*-GlcNAc-Asn (6′FN-Asn), a constituent of the core-fucosylated structures of mammalian *N*-glycoproteins. The cluster consists of the genes *alfHC*, encoding a major facilitator superfamily (MFS) permease and the α-l-fucosidase AlfC, and the divergently oriented *asdA* (aspartate 4-decarboxylase), *alfR2* (transcriptional regulator), *pepV* (peptidase), *asnA2* (glycosyl-asparaginase), and *sugK* (sugar kinase) genes. Knockout mutants showed that *alfH*, *alfC*, *asdA*, *asnA2*, and *sugK* are necessary for efficient 6′FN-Asn utilization. The *alf-2* genes are induced by 6′FN-Asn, but not by its glycan moiety, via the AlfR2 regulator. The constitutive expression of *alf-2* genes in an *alfR2* strain allowed the metabolism of a variety of 6′-fucosyl-glycans. However, GlcNAc-Asn did not support growth in this mutant background, indicating that the presence of a 6′-fucose moiety is crucial for substrate transport via AlfH. Within bacteria, 6′FN-Asn is defucosylated by AlfC, generating GlcNAc-Asn. This glycoamino acid is processed by the glycosylasparaginase AsnA2. GlcNAc-Asn hydrolysis generates aspartate and GlcNAc, which is used as a fermentable source by L. casei. These data establish the existence in a commensal bacterial species of an exclusive metabolic pathway likely to scavenge human milk and mucosal fucosylated *N*-glycopeptides in the gastrointestinal tract.

## Abstract (precis)

The gastrointestinal tract accommodates more than 10^14^ microorganisms that have an enormous impact on human health. The mechanisms enabling commensal bacteria and administered probiotics to colonize the gut remain largely unknown. The ability to utilize host-derived carbon and energy resources available at the mucosal surfaces may provide these bacteria with a competitive advantage in the gut. Here, we have identified in the commensal species Lactobacillus casei a novel metabolic pathway for the utilization of the glycoamino acid fucosyl-α-1,6-*N*-GlcNAc-Asn, which is present in the core-fucosylated *N*-glycoproteins from mammalians. These results give insight into the molecular interactions between the host and commensal/probiotic bacteria and may help to devise new strategies to restore gut microbiota homeostasis in diseases associated with dysbiotic microbiota.

## INTRODUCTION

Many investigations have recently highlighted the importance of the gut microbiota in the onset and progression of a number of human diseases, including gastrointestinal disorders (1, 2), inflammatory diseases (3), respiratory tract infections (4), and allergies (5). The functional impact of commensal gut microorganisms depends on their ability to survive in the gastrointestinal tract, adhere to epithelial mucus, and obtain energy from nondigestible dietary substrates and host mucosal secretions (6). More than half of all proteins in nature have been estimated to be glycosylated through *O*-glycosidic or *N*-glycosidic bonds (7). *O*-Glycans are linked to a serine or threonine residue via an *N*-acetylgalactosamine, which is elongated by additional sugars (8). *N*-Glycosylation is a common modification of extracellular membrane proteins present at the gastrointestinal epithelium, the secreted proteins of human breast milk, and many dietary proteins (7, 9–11). *N*-Linked glycans are attached via the core *N*,*N*′-diacetylchitobiose disaccharide (ChbNAc) (GlcNAc-β1,4-GlcNAc) to an asparagine residue of proteins containing the Asn-Xxx-Ser/Thr (with Xxx being any amino acid except Pro) motif (12). In *N*-glycans from mammals, the inner GlcNAc moiety bound to Asn is often fucosylated through an α1,6 linkage, named core fucose. Protein *N*-glycosylation plays a crucial role in a variety of cellular processes, such as cell adhesion (13), immune pathway signaling (14), and bacterial recognition (15). Some intestinal microorganisms have the ability to process the carbohydrate moieties of *N*-glycosylated proteins (16) so that the type, abundance, and location of these glycans contribute to shaping the composition and distribution of the gut microbiota (17). Some bacterial pathogens possess endo-β-*N*-acetylglucosaminidase enzymes that cleave the β-1,4 linkage of the core ChbNAc present in all *N*-glycoproteins, releasing the *N*-glycan moiety (18). The activities of these enzymes have been associated with the modification of the biological function of host defense glycoproteins, such as immunoglobulins and lactoferrin (19–21), and with the use of the glycans as nutrients (21), for which they are considered virulence factors (22). In commensal bacteria, the ability to remove *N*-glycans from glycoproteins has been described in a few *Bifidobacterium* species (23, 24). Recently, the importance of core-fucosylated *N*-glycans from human milk in promoting the intestinal growth of *Bifidobacterium* and *Lactobacillus* species has been demonstrated in lactating infants from mothers carrying different alleles of the fucosyltransferase Fut8, responsible for core fucosylation (25). This provides the first *in vivo* evidence of the importance of this core structure in feeding intestinal commensals. However, there is little information about the fate of the fucosyl-α-1,6-GlcNAc bound to proteins through the Asn residue (6′FN-Asn). This glycoamino acid possibly results from the combined action of endo-β-*N*-acetylglucosaminidase enzymes and proteases on *N*-glycosylated proteins (18, 23, 24). The amide bond between the amino acid and the GlcNAc residue is hydrolyzed by two different enzymes, peptide-*N*(4)-(β-*N*-acetylglucosaminyl)-l-asparaginases (glycopeptide *N*-glycosidase) (PNGase) (EC 3.5.1.52) and *N*(4)-(β-*N*-acetylglucosaminyl)-l-asparaginases (glycosylasparaginase) (EC 3.5.1.26). Both types of enzymes are produced as precursors that undergo intramolecular autoproteolysis to produce the mature active proteins (26, 27), but PNGases require the presence of more than 2 amino acid residues in the substrate (28), whereas glycosylasparaginases act only in asparagine-oligosaccharides containing 1 amino acid (29). Currently, bacterial PNGases have been characterized only from the human pathogens Elizabethkingia meningoseptica and Elizabethkingia miricola (30, 31) and from the soil bacterium Terriglobus roseus (32). In E. meningoseptica, a glycosylasparaginase has also been characterized (33, 34).

Lactobacillus casei is a lactic acid bacterium able to survive in the gastrointestinal tract (35, 36), which has been isolated from a wide variety of habitats, including feces of breastfed infants (37, 38), and several strains are commonly used as probiotics in functional foods (39, 40). Oligosaccharides present in human milk, such as lacto-*N*-biose and *N*-acetyllactosamine, and derived from mucins, such as galacto-*N*-biose, can support the growth of L. casei (41, 42). This species is also able to catabolize lacto-*N*-triose (43) and fucosyl-α-1,3-*N-*acetylglucosamine (44), which are abundant carbohydrates that form part of larger glycan structures from human gut mucosas and human milk. Unlike glycans, catabolic pathways for *N*-glycopeptides in bacteria have not been described. In this work, we have identified in *L. casei* BL23 a gene cluster, named *alf-2*, involved in the metabolism of the glycoamino acid fucosyl-α-1,6-*N*-GlcNAc-Asn (6′FN-Asn). The results reported here have enabled us to propose a catabolic pathway for 6′FN-Asn and α-1,6-fucosylated *N*-glycans in bacteria.

## RESULTS

### Identification of the *L. casei alf-2* gene cluster involved in the metabolism of the glycoamino acid 6′FN-Asn.

**FIG 1:**
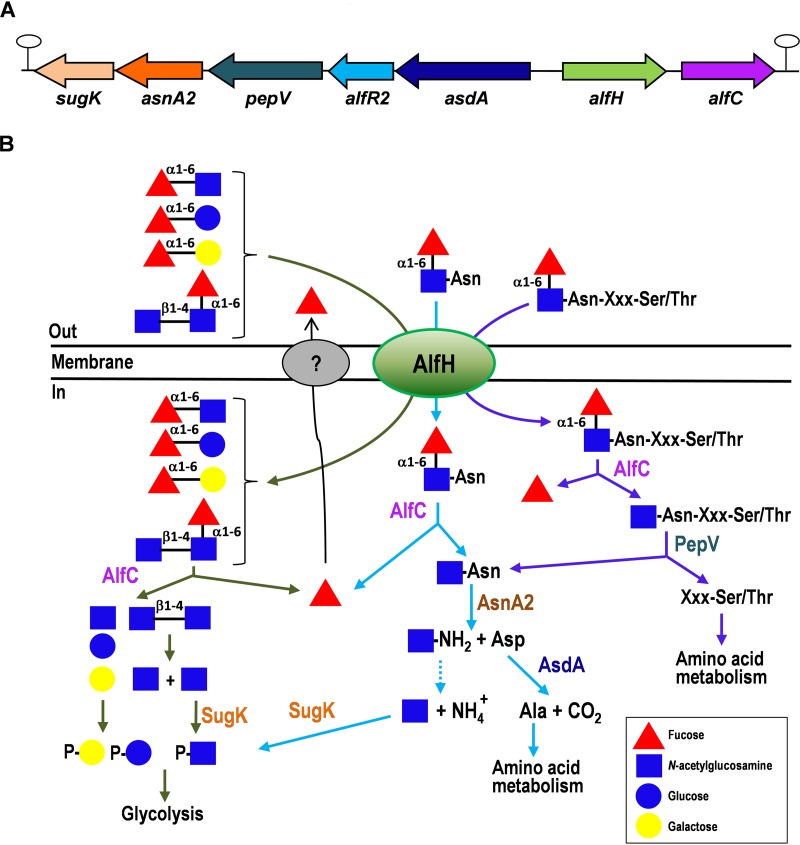
(A) Structural organization of the Lactobacillus casei BL23 *alf-2* operon. (B) Schematic representation of the transport and catabolic pathways for fucosyl-α-1,6-*N-*acetylglucosamine-asparagine, fucosylated glycans (fucosyl-α-1,6-*N-*acetylglucosamine, fucosyl-α-1,6-glucose, fucosyl-α-1,6-galactose, and fucosyl-α-1,6-*N*,*N′*-diacetylchitobiose), and fucosylated *N*-glycopeptides in Lactobacillus casei. AlfC, α-l-fucosidase AlfC; AsnA2, *N*(4)-(β-*N*-acetylglucosaminyl)-l-asparaginase; AsdA, aspartate 4-decarboxylase; PepV, peptidase V; SugK, sugar kinase.

**FIG 2:**
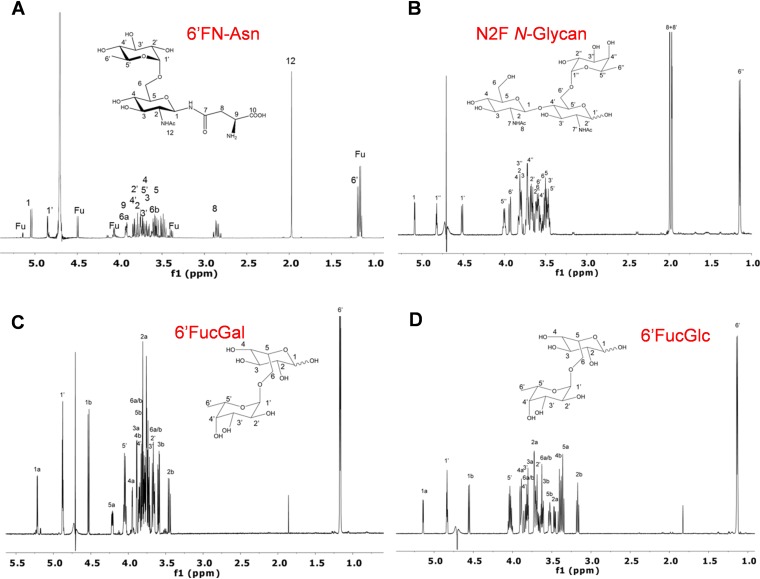
^1^H NMR spectra of compounds in D_2_O acquired at 27°C at 600 MHz with an inverse cryoprobe. (A) Fucosyl-α-1,6-*N*-acetylglucosamine-asparagine (6′FN-Asn); (B) fucosyl-α-1,6-*N*,*N′*-diacetylchitobiose (N2F *N*-glycan); (C) fucosyl-α-1,6-galactose (6′FucGal); (D) fucosyl-α-1,6-glucose (6′FucGlc). Signals labeled with Fu correspond to copurified l-fucose.

We had previously shown that the disaccharide fucosyl-α-1,6-*N-*acetylglucosamine (6′FN) is hydrolyzed *in vitro* by the *L. casei* BL23 α-l-fucosidase AlfC (glycosyl hydrolase family 29 [GH29]) (45). However, this bacterium is unable to grow in the presence of 6′FN as a carbon source (44). Analysis of the DNA region (GenBank accession no. FM177140) (46) around *alfC* revealed a gene cluster named here *alf-2* (Fig. 1A). This cluster consists of the genes *alfHC* (LCABL_RS14345 and LCABL_RS14350), which encode a major facilitator superfamily (MFS) permease and AlfC, respectively, and, divergently oriented, the genes *asdA* (LCABL_RS14340), *alfR2* (LCABL_RS14335), *pepV* (LCABL_RS14330), *asnA2* (LCABL_RS14325), and *sugK* (LCABL_RS14320). These genes encode proteins annotated as aspartate 4-decarboxylase (*asdA*), a GntR family transcriptional regulator (*alfR2*), peptidase M20 (*pepV*), *N*(4)-(β-*N*-acetylglucosaminyl)-l-asparaginase (*asnA2*), and a ROK (repressor, open reading frame [ORF], kinase) family protein (*sugK*). Two putative *rho*-independent terminators were identified downstream of *alfC* (Δ*G*, −13.5 kcal/mol) and *sugK* (Δ*G*, −17.0 kcal/mol). The high specificity of the α-l-fucosidase AlfC from *L. casei* BL23 for α1,6 linkages such as those present at fucosyl-oligosaccharides (45, 47), together with the sequence analysis of the *alf-2* cluster, particularly the presence of a gene coding for a hypothetical *N*(4)-(β-*N*-acetylglucosaminyl)-l-asparaginase, suggested that the *alf-2* operon could be involved in the metabolism of the core 6′FN-Asn (Fig. 1). To test this hypothesis, this glycoamino acid was synthesized by transfucosylation with the α-l-fucosidase AlfC. We had previously utilized AlfC in transglycosylation reactions with *p*-nitrophenyl-α-l-fucopyranoside (*p*NP-fuc) as the donor and GlcNAc as the acceptor to produce 6′FN (47). Here, the ability of AlfC to use GlcNAc-Asn as the acceptor was tested, and 6′FN-Asn was synthesized and purified. The purified 6′FN-Asn was characterized by nuclear magnetic resonance (NMR) spectroscopy (Fig. 2A) (see Fig. S1 and Table S1 in the supplemental material). ChbNAc, galactose, and glucose were also used in transfucosylation reactions with AlfC, and the glycans fucosyl-α-1,6-*N*,*N*′-diacetylchitobiose (N2F *N*-glycan), which forms part of the core fucosylation; fucosyl-α-1,6-galactose (6′FucGal); and fucosyl-α-1,6-glucose (6′FucGlc) were also synthesized (Fig. 2B to D and Table S1).

10.1128/mBio.02804-19.1 FIG S1 Zoom of the 2D HMBC NMR spectrum of the compound fucosyl-α-1,6-*N*-acetylglucosamine-asparagine in D_2_O acquired at 27°C at 600 MHz with an inverse cryoprobe. The correlation between C1′ and H6a and H6b clearly reflects the binding of 1,6 between both sugar molecules. Download FIG S1, TIF file, 0.7 MB. Copyright © 2020 Becerra et al. 2020 Becerra et al. This content is distributed under the terms of the Creative Commons Attribution 4.0 International license.

10.1128/mBio.02804-19.7 TABLE S1 ^1^H and ^13^C assignments of the compounds fucosyl-α-1,6-*N*-acetylglucosamine-asparagine, fucosyl-α-1,6-*N*,*N*′-diacetylchitobiose, fucosyl-α-1,6-galactose, and fucosyl-α-1,6-glucose carried out at 27°C at 600 MHz in D_2_O. The spectrum was referenced to the water signal at 4.7 ppm. Download Table S1, DOCX file, 0.02 MB. Copyright © 2020 Becerra et al. 2020 Becerra et al. This content is distributed under the terms of the Creative Commons Attribution 4.0 International license.

**FIG 3:**
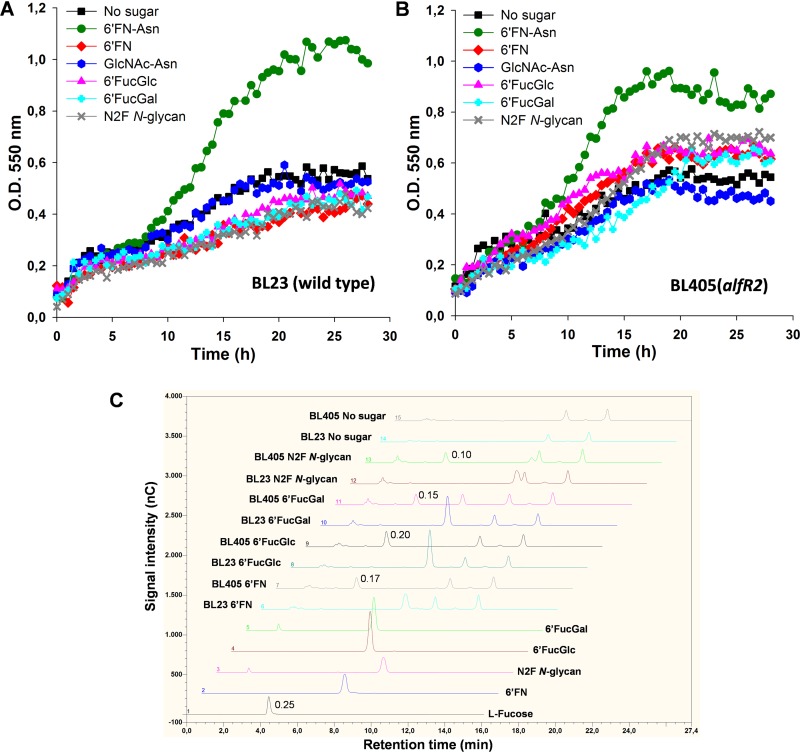
Growth profiles of Lactobacillus casei on *N*-glycan derivatives. (A and B) *L. casei* wild-type strain BL23 (A) and strain BL405, an *alfR2* deletion mutant (B), grown on MRS basal medium without a carbon source or with fucosyl-α-1,6-*N-*acetylglucosamine-asparagine (6′FN-Asn), fucosyl-α-1,6-*N-*acetylglucosamine (6′FN), *N*-acetylglucosamine-asparagine (GlcNAc-Asn), fucosyl-α-1,6-glucose (6′FucGlc), fucosyl-α-1,6-galactose (6′FucGal), or fucosyl-α-1,6-*N*,*N*′-diacetylchitobiose (N2F *N*-glycan). (C) HPLC chromatograms (Dionex system) of the standard compounds l-fucose (0.25 mM) (chromatogram 1), 6′FN (0.2 mM) (chromatogram 2), N2F *N*-glycan (0.2 mM) (chromatogram 3), 6′FucGlc (0.2 mM) (chromatogram 4), and 6′FucGal (0.2 mM) (chromatogram 5) and culture supernatants (diluted 20 times) from *L. casei* BL23 (WT) (chromatograms 6, 8, 10, 12, and 14) and BL405 (*alfR2*) (chromatograms 7, 9, 11, 13, and 15) grown in 6′FN (chromatograms 6 and 7), 6′FucGlc (chromatograms 8 and 9), 6′FucGal (chromatograms 10 and 11), or N2F *N*-glycan (chromatograms 12 and 13) or without sugar (chromatograms 14 and 15). The numbers by the l-fucose peaks indicate the concentration (millimolar). nC, nanoCoulomb.

To determine whether *L. casei* BL23 is able to use 6′FN-Asn, GlcNAc-Asn, N2F *N*-glycan, 6′FucGal, or 6′FucGlc as a carbon source, its growth profiles in MRS basal medium supplemented with each compound were analyzed. *L. casei* grew only in the presence of the glycoamino acid 6′FN-Asn (Fig. 3A). The analysis of the carbohydrate content of the growth medium showed that BL23 did not degrade any of the assayed glycans (Fig. 3C). However, the AlfR2-deficient strain (BL405) could degrade all the synthesized 6′-fucosyl oligosaccharides, as evidenced by the quantitative accumulation of l-fucose in the supernatant and the disappearance of the peaks corresponding to the fucosylated substrates (Fig. 3C). The presence of l-fucose in the culture supernatants was due to the fact that *L. casei* does not metabolize l-fucose and excretes it into the culture medium (44). Nevertheless, growth with these carbon sources was very poor compared to that with 6′FN-Asn (Fig. 3B). Interestingly, like the wild type (WT), the *alfR2* mutant strain did not use GlcNAc-Asn (Fig. 3B). These results suggested that AlfR2 represses the expression of *alf-2* genes and that the presence of 6′FN-Asn is required to relieve repression. Furthermore, the existence of an l-fucose moiety with an α1,6 linkage configuration is possibly necessary for the uptake of the tested fucosyl oligosaccharides, including 6′FN-Asn.

### Transcription of the *alf-2* gene cluster is induced by 6′FN-Asn and repressed by AlfR2.

**FIG 4:**
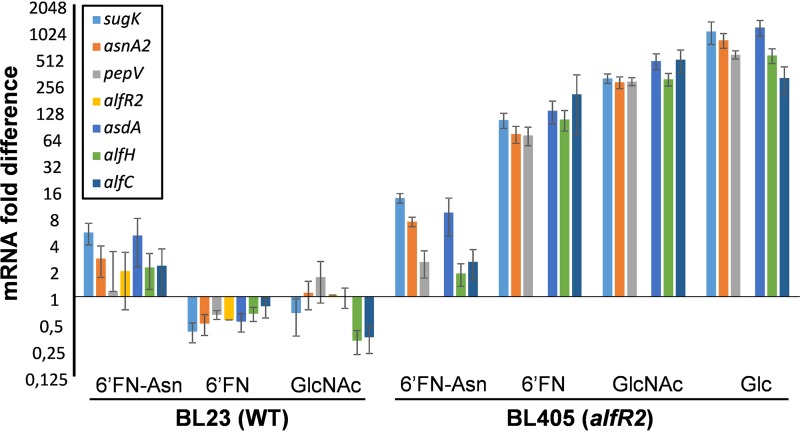
Expression of *alf-2* genes in Lactobacillus casei. *L. casei* BL23 (WT) and *L. casei* BL405 (*alfR2* deletion mutant) were grown in MRS basal medium containing fucosyl-α-1,6-*N-*acetylglucosamine-asparagine (6′FN-Asn), fucosyl-α-1,6-*N-*acetylglucosamine (6′FN), or *N*-acetylglucosamine (GlcNAc). Gene expression in *L. casei* BL405 in the presence of glucose is also shown. Expression was monitored by RT-qPCR, and *L. casei* BL23 (WT) cells grown in MRS basal medium with glucose were used as a reference. Data presented are mean values based on at least three replicates. Bars indicate standard errors.

To find out whether the transcription of the *alf-2* genes is regulated by the glycoamino acid 6′FN-Asn or its glycan moiety, reverse transcription-quantitative PCR (RT-qPCR) experiments were performed with RNA isolated from *L. casei* BL23 grown with 6′FN-Asn, 6′FN, GlcNAc, or glucose (Fig. 4). The results showed that the *alf-2* operon is induced by the glycoamino acid 6′FN-Asn and not by the presence of 6′FN or GlcNAc, indicating that the glycoamino acid and not the glycan moiety is responsible for the induction of these genes and explaining the lack of growth of the wild type on N2F *N*-glycan, 6′FucGal, and 6′FucGlc. In order to obtain direct evidence for the regulation of AlfR2 on these genes, RNA was isolated from the *alfR2* deletion strain BL405 cultured under the same conditions as those for the wild type (Fig. 4). Regardless of the substrate tested, the expression levels of *alf-2* genes were higher than those in the wild type growing on glucose, indicating that AlfR2 indeed acts as a transcriptional repressor.

### The AlfC α-l-fucosidase and the AlfH permease are necessary for the metabolism of 6′FN-Asn and 6′-fucosyl-oligosaccharides.

**FIG 5:**
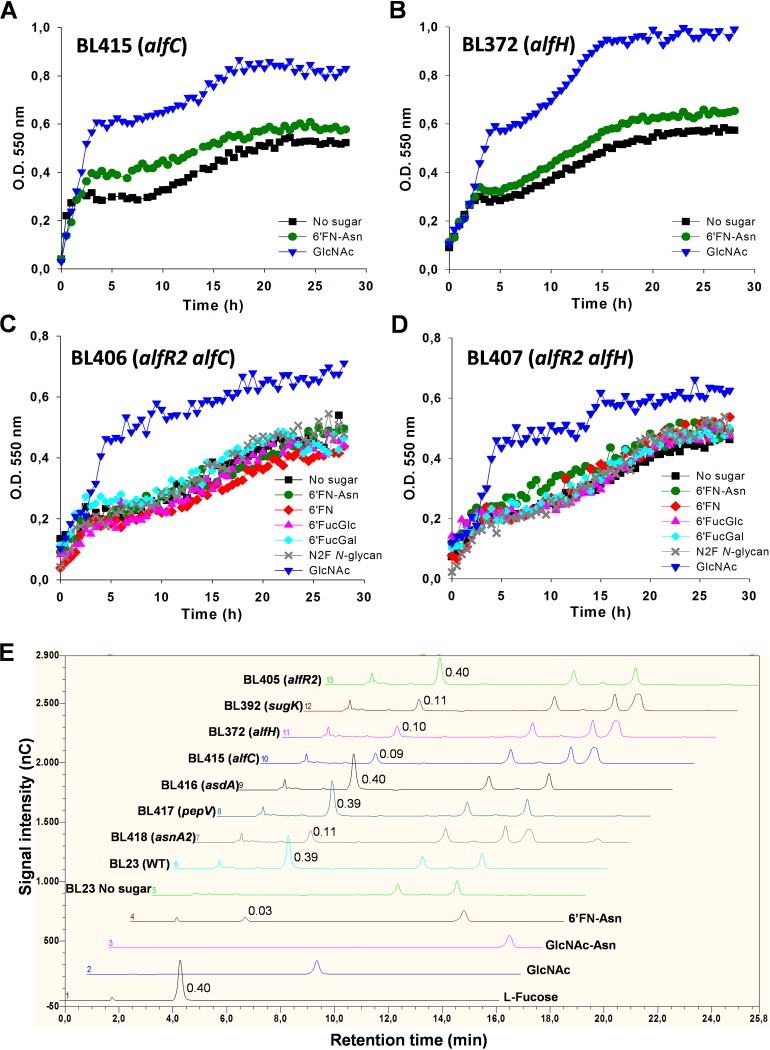
Growth curves of Lactobacillus casei mutant strains. (A and B) BL415 (*alfC*) (A) and BL372 (*alfH*) (B) on MRS basal medium without a carbon source or with fucosyl-α-1,6-*N-*acetylglucosamine-asparagine (6′FN-Asn) or *N-*acetylglucosamine (GlcNAc). (C and D) BL406 (*alfR2 alfC*) (C) and BL407 (*alfR2 alfH*) (D) on MRS basal medium without a carbon source or with 6′FN-Asn, fucosyl-α-1,6-*N-*acetylglucosamine (6′FN), fucosyl-α-1,6-glucose (6′FucGlc), fucosyl-α-1,6-galactose (6′FucGal), fucosyl-α-1,6-*N*,*N*′-diacetylchitobiose (N2F *N*-glycan), or GlcNAc. The four mutant strains were grown in MRS basal medium with GlcNAc as a positive control. (E) HPLC chromatograms (Dionex system) of the supernatants (diluted 10 times) from Lactobacillus casei strain cultures. Shown are chromatograms of the standards l-fucose (0.4 mM) (chromatogram 1), GlcNAc (0.1 mM) (chromatogram 2), *N-*acetylglucosamine-asparagine (GlcNAc-Asn) (0.1 mM) (chromatogram 3), and 6′FN-Asn (0.1 mM) (chromatogram 4). Also shown are chromatograms of the culture supernatants from *L. casei* BL23 (WT) cultured without added sugar (chromatogram 5) or from *L. casei* strains cultured on 6′FN-Asn, including BL23 (WT) (chromatogram 6), BL418 (*asnA2*) (chromatogram 7), BL417 (*pepV*) (chromatogram 8), BL416 (*asdA*) (chromatogram 9), BL415 (*alfC*) (chromatogram 10), BL372 (*alfH*) (chromatogram 11), BL392 (*sugK*) (chromatogram 12), and BL405 (*alfR2*) (chromatogram 13). The numbers by the l-fucose peaks indicate the concentration (millimolar). nC, nanoCoulomb.

To assess the role of *alfC* and *alfH* in the catabolism of 6′FN-Asn in *L. casei*, the growth patterns of mutant strains disrupted in *alfC* (BL415) and *alfH* (BL372) were analyzed in MRS basal medium supplemented with the fucosylated glycoamino acid (Fig. 5A and B). The profiles showed that both mutants failed to grow on 6′FN-Asn. To further confirm that the glycoamino acid was not fermented, culture supernatant analyses were performed, and they showed that 6′FN-Asn remained in BL415 and BL372 supernatants, without the appearance of l-fucose (Fig. 5E). The amount of l-fucose (1.12 mM) present in both supernatants corresponded to the amount of l-fucose copurified with the synthesized 6′FN-Asn (Fig. 2 and Fig. 5E). These results demonstrated that the α-l-fucosidase AlfC is involved in the metabolism of 6′FN-Asn and that the permease AlfH participates in its transport. To determine the role of AlfC and AlfH in the utilization of the rest of the fucosyl-glycans synthesized here (6′FN, 6′FucGlc, 6′FucGal, and N2F *N*-glycan), two double mutant strains, BL406 (*alfR2 alfC*) and BL407 (*alfR2 alfH*), were constructed. Both mutants showed growth patterns with 6′-fucosyl-glycans similar to that of the negative-control culture without an added carbohydrate (Fig. 5C and D). In addition, the results showed a final culture optical density (OD) of strain BL406 or BL407 that was significantly lower than that of strain BL405 (*alfR2*) on these sugars (Table S2). These results indicated that the α-l-fucosidase AlfC and the permease AlfH are also involved in their metabolism and transport, respectively. 6′FN-Asn was also tested as a carbon source in the culture medium with these double mutants, confirming the requirement of a functional α-l-fucosidase and permease for its metabolism (Fig. 5C and D).

10.1128/mBio.02804-19.8 TABLE S2 Final OD values (means ± standard deviations) reached by Lactobacillus casei mutant strains cultured on 6′-fucosyl-glycans. Download Table S2, DOCX file, 0.02 MB. Copyright © 2020 Becerra et al. 2020 Becerra et al. This content is distributed under the terms of the Creative Commons Attribution 4.0 International license.

### 6′FN-Asn catabolism requires AsdA, AsnA2, and SugK but not PepV.

**FIG 6:**
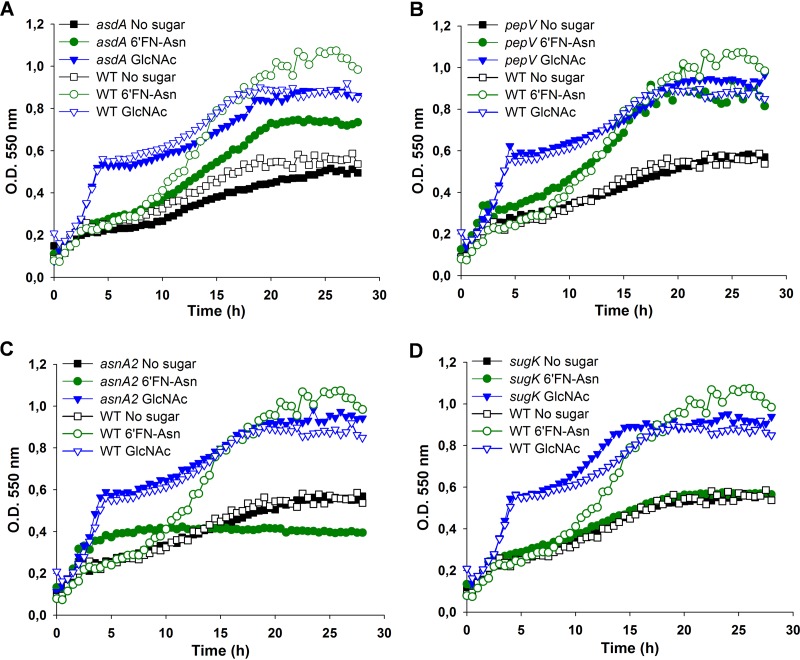
Growth curves of Lactobacillus casei mutant strains. Shown are growth curves for BL416 (*asdA*) (A), BL417 (*pepV*) (B), BL418 (*asnA2*) (C), and BL392 (*sugK*) (D) on MRS basal medium without a carbon source or with fucosyl-α-1,6-*N-*acetylglucosamine-asparagine (6′FN-Asn) or *N-*acetylglucosamine (GlcNAc). In all graphs, the growth pattern of wild-type (WT) strain BL23 is presented for a better comparison.

An *asdA* mutant strain (BL416) showed impaired growth in MRS basal medium supplemented with 6′FN-Asn (Fig. 6A). Curiously, the glycoamino acid was completely depleted from the culture supernatant (Fig. 5E); however, the mutant strain reached a lower optical density than the wild-type strain, suggesting that *asdA* is involved in 6′FN-Asn metabolism. A BLAST search using the deduced amino acid sequence of AsdA against the genomic sequence of *L. casei* BL23 did not reveal the presence of other AsdA paralogues. AsdA belongs to the Asp aminotransferase family (cd00609), which also includes a number of enzymes with decarboxylase or racemase activities. The BL23 genome encoded 11 hypothetical carboxylases (https://www.ncbi.nlm.nih.gov/genome/proteins/652?genome_assembly_id=300266); whether AsdA is a decarboxylase and whether any of these enzymes would complement its activity need to be investigated. A *pepV* mutant strain (BL417) showed a growth pattern similar to that of the wild-type strain (Fig. 6B), indicating that the hypothetical peptidase encoded by this gene is not essential for the metabolism of 6′FN-Asn. The two genes *asnA2* and *sugK*, present downstream of *pepV*, were, however, necessary for the utilization of 6′FN-Asn by *L. casei*. An *asnA2* (BL418) mutant strain constructed here and a *sugK* (BL392) mutant characterized previously (41) failed to grow on 6′FN-Asn (Fig. 6C and D). These results indicate that both the putative glycosylasparaginase AsnA2 and the putative sugar kinase SugK are required for the catabolism of 6′FN-Asn.

### AsnA2 has glycosylasparaginase activity.

The capacity to catabolize 6′FN-Asn by *L. casei* indicated that AsnA2 probably encodes a glycosylasparaginase that would cleave the linkage between GlcNAc and Asn. To confirm this hypothesis, AsnA2 was overexpressed as a His-tagged protein and purified (Fig. S2). The molecular weight of His-tagged AsnA2 was estimated to be 55.6 kDa using size exclusion chromatography, which does not coincide with the theoretical molecular mass (36.071 kDa), suggesting that the enzyme probably forms dimers. Interestingly, when the purified AsnA2 fraction was subjected to SDS-PAGE analysis, three bands were observed: a very faint band at about 38.0 kDa and two other bands with estimated molecular weights of 18.2 and 17.8 kDa. Mass spectrometry (MS) analysis showed that all three protein bands were derived from the glycosylasparaginase AsnA2 (data not shown). The 38-kDa band corresponds to the full-length protein, whereas the 18.2- and 17.8-kDa fragments correspond to the N-terminal and C-terminal domains, respectively. Therefore, AsnA2 is probably a zymogen that is processed during purification. A mechanism of intramolecular autoproteolysis has been previously described for glycosylasparaginases and PNGases (26, 27). The self-processing of the precursor protein occurs at a Thr residue, and the two fragments form a noncovalent heterodimeric complex (26). A BLAST search with the amino acid sequence of *L. casei* AsnA2 evidenced 28% sequence identity to the glycosylasparaginase of *E. meningoseptica* (26). The sequence alignment between both proteins revealed that all residues involved in autoproteolytic processing, including the catalytic Thr residue (Thr-154 in AsnA2), are conserved. We analyzed the activity of the purified AsnA2 by measuring the release of GlcNAc from GlcNAc-Asn, which confirmed that AsnA2 is an *N*(4)-(β-*N*-acetylglucosaminyl)-l-asparaginase (Fig. S2). This exhibited a specific activity of 20.91 μmol mg protein^−1^ min^−1^ for GlcNAc-Asn. The activity of AsnA2 on the fucosylated glycoamino acid was also tested (Fig. S2). In contrast to the human glycosylasparaginase, which does not act on 6′FN-Asn (48), AsnA2 was able to degrade 6′FN-Asn, releasing 6′FN with a specific activity of 0.26 μmol mg protein^−1^ min^−1^. These results indicated that AsnA2 preferentially acts on GlcNAc-Asn over the fucosylated substrate 6′FN-Asn.

10.1128/mBio.02804-19.2 FIG S2 (A) SDS-polyacrylamide gel electrophoresis (PAGE) analysis of the protein AsnA2. SDS-PAGE was performed in 12% gels under reducing conditions, and the proteins were Coomassie blue stained. Lane P, protein standards. The numbers on the left are molecular masses. The arrows on the right point to the AsnA2 protein bands. (B) Chromatogram showing size exclusion chromatography of AsnA2. All products elute as a single peak with an estimated molecular weight of 55.6 kDa. (C) Calibration plot of standard proteins. Gel filtration experiments with five standard proteins were used. (D) Deglycosylation assays. SDS-PAGE analysis of ovalbumin (OVA) and lactoferrin (LAC) without (−) and with (+) the glycosylasparaginase AsnA2 was performed. SDS-PAGE was performed with 10% gels under reducing conditions, and the proteins were Coomassie blue stained. Lane P, protein standards. The numbers on the right are molecular masses. (E) HPLC chromatograms (Dionex system) of reaction mixtures containing 0.2 μg of the AsnA2 enzyme, 100 mM Tris-HCl buffer (pH 7.0), and 5 mM *N*-acetylglucosamine-asparagine (GlcNAc-Asn) (chromatograms 5, 6, and 7 correspond to reaction times of 0.5, 2, and 4 min, respectively) or fucosyl-α-1,6-*N*-acetylglucosamine-asparagine (6′FN-Asn) (chromatograms 8, 9, and 10 correspond to reaction times of 0.5, 2, and 4 min, respectively). Each reaction mixture was diluted 20 times before HPLC analysis. The red arrows show the *N*-acetylglucosamine (GlcNAc) released by AsnA2 from GlcNAc-Asn, and the blue arrows show the fucosyl-α-1,6-*N*-acetylglucosamine (6′FN) released from 6′FN-Asn in the zoomed image. Chromatograms of the standards at 0.1 mM are GlcNAc (chromatogram 1), GlcNAc-Asn (chromatogram 2), 6′FN (chromatogram 3), and 6′FN-Asn (chromatogram 4). Download FIG S2, TIF file, 1.6 MB. Copyright © 2020 Becerra et al. 2020 Becerra et al. This content is distributed under the terms of the Creative Commons Attribution 4.0 International license.

### AsdA displays aspartate 4-decarboxylase activity, and SugK displays kinase activity.

As described below, the Asp resulting from the activity of AsnA2 on GlcNAc-Asn might be the substrate for the AsdA enzyme. To test this hypothesis, AsdA was overexpressed with a His tag and purified. Recombinant AsdA showed decarboxylation activity on l-Asp, with a specific activity of 10.97 ± 2.27 nmol mg protein^−1^ min^−1^. This weak activity on l-Asp might indicate that this amino acid is not the preferred substrate for AsdA. Regarding SugK, the proposed pathways for 6′FN-Asn and 6′-fucosyl-glycans (Fig. 1B) involved the release of the monosaccharides GlcNAc, Glc, and Gal, which should be phosphorylated by specific kinases before entering glycolysis. In order to prove whether SugK showed kinase activity on these sugars, it was overexpressed as a His-tagged protein and purified. SugK displayed kinase activity on GlcNAc (1.44 μmol mg protein^−1^ min^−1^) but could not phosphorylate Glc and Gal. Activity on *N*-acetylgalactosamine (GalNAc) was also assayed, as this *N*-acetylhexosamine is also very abundant on mucosa-associated glycans, and no activity on this sugar was detected.

### Proposed pathway for 6′FN-Asn catabolism in *L. casei*.

The genetic and biochemical evidence reported here allows the proposal of a catabolic pathway for 6′FN-Asn in *L. casei* (Fig. 1B). The glycoamino acid is internalized by the permease AlfH, which has broad specificity over a range of 6′-fucosylated substrates, and is subsequently defucosylated by the α-l-fucosidase AlfC, generating l-fucose and GlcNAc-Asn. The released l-fucose is excreted from the cells by an as-yet-undetermined mechanism, while GlcNAc-Asn is split by AsnA2 into Asp and 1-amino-GlcNAc, which is unstable, and it is nonenzymatically converted to GlcNAc and ammonium (49, 50). The requirement of AlfC for 6′FN-Asn utilization and the lower affinity of AsnA2 for 6′FN-Asn than for GlcNAc-Asn support that the catabolism of 6′FN-Asn occurs through the consecutive actions of AlfC and AsnA2. GlcNAc produced by the action of AsnA2 on GlcNAc-Asn would then be the substrate for the sugar kinase SugK (Fig. 1). The other resulting product of AsnA2 activity, Asp, might be the substrate of AsdA, although the weak aspartate 4-decarboxylase activity could indicate that AsdA may play an as-yet-undetermined role in this pathway. Regarding the function of *pepV*, which encodes a putative peptidase, no signal peptide was evidenced for it, suggesting that it is a cytoplasmic protein. The permease AlfH might also transport more complex substrates, like *N*-glycosylated peptides derived from the proteolysis of host- and food-derived proteins. Furthermore, the fact that AlfC is able to release l-fucose from core fucosylation of the Fc fragment from immunoglobulins (51) shows that this enzyme has the capacity to hydrolyze α-1,6 linkages in polypeptidic substrates and could act on core-fucosylated *N*-glycopeptides. In this case, after the release of l-fucose by AlfC, amino acids would be removed by PepV to liberate GlcNAc-Asn (Fig. 1B).

## DISCUSSION

*N*-Glycosylated proteins are present at human mucosal surfaces and in breast milk (9, 10, 52), and therefore, they can be accessible to the gut microbiota. Most studies about energy sources for gut-beneficial microbes have been focused on carbohydrates present in the diet or added as prebiotics (53), whereas knowledge about the catabolism of host-derived carbon and nitrogen sources is scarce (16). We have demonstrated that *L. casei* is able to metabolize the glycoamino acid 6′FN-Asn, which is the core structure of the *N*-glycan sites of α1,6-fucosylated glycoproteins. The relevance of the metabolism of this core structure for intestinal microbial ecology was recently established *in vivo* in humans and in mouse models with reduced α1,6-fucosylation of core structures (25). In this work, we filled the existing gap relative to the metabolic pathways involved in the utilization of core-fucosylated *N*-glycopeptides by bacteria. The backbone of the *L. casei* 6′FN-Asn pathway consists of the MFS transporter AlfH; the α-l-fucosidase AlfC, which removes the α1,6-fucosyl residue; and the glycosylasparaginase AsnA2, which processes the resulting GlcNAc-Asn to 1-amino-GlcNAc and Asp (Fig. 1B). The generated 1-amino-GlcNAc is not metabolizable, but this compound is unstable at acidic pH, and it degrades into GlcNAc and ammonium (49). As lactobacilli do not maintain a constant internal pH, but it decreases as the external pH drops (54), intracellular conditions may allow the spontaneous degradation of 1-amino-GlcNAc.The resulting GlcNAc would then be the substrate for the sugar kinase SugK, allowing its channeling through glycolysis.

**FIG 7:**
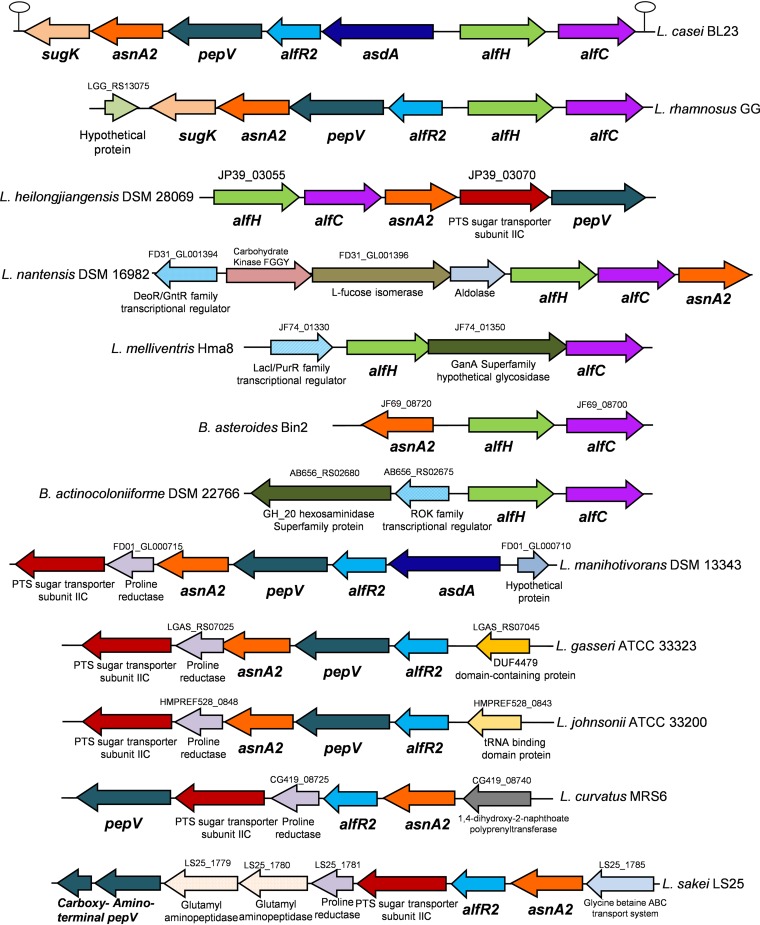
Structural organization of the *alf-2* gene clusters from Lactobacillus rhamnosus GG, Lactobacillus heilongjiangensis DSM 28069, Lactobacillus nantensis DSM 16982, Lactobacillus melliventris Hma8, Bifidobacterium asteroides Bin2, Bifidobacterium actinocoloniiforme DSM 22766, Lactobacillus manihotivorans DSM 13343, Lactobacillus gasseri ATCC 33323, Lactobacillus johnsonii ATCC 33200, Lactobacillus curvatus MRS6, and Lactobacillus sakei LS25. Stem-loop structures in *L. casei* DNA represent putative *rho*-independent terminators. Surrounding genes near the *alf-2* genes are also shown. The organization of the Lactobacillus casei BL23 *alf-2* operon is also shown for a better comparison. PTS, phosphotransferase system.

The genetic organization of the *L. casei alf-2* gene cluster is well conserved in gene content and gene order across the *L. casei*-Lactobacillus paracasei-Lactobacillus rhamnosus phylogenetically related group of lactobacilli, excepting the absence of *asdA* in L. rhamnosus (Fig. 7). Homologues of *alfC* are present in only a few *Lactobacillus* species, and the closest homologues are harbored by some bifidobacteria isolated from Hymenoptera (55–57) (see Fig. S3A in the supplemental material). Interestingly, many lactobacilli carrying *alfC* have also been isolated from insects (58, 59). Phylogenetic analyses of these genes show that they constitute a well-supported cluster together with the *alfC* gene of Vagococcus humatus. More distant homologues are mostly present in gut anaerobic bacteria (Fig. S3A). Homologues of *L. casei alfH* are mostly found in the same set of species, although this gene is also present in other bifidobacteria and in Lactobacillus kisonensis (Fig. S3B). The genetic association of these genes in these species and their close phylogenetic relationships suggest that they share an origin and an evolutionary history. Homologues of AsnA2 (Fig. S4), AsdA (Fig. S5), and PepV (Fig. S6) are present in numerous lactobacilli. Indeed, Lactobacillus gasseri ATCC 33323, which carries a gene cluster with *asnA2*, *pepV*, and *alfR2* (Fig. 7), was recently shown to grow with 6′FN and N2F *N*-glycan (25). However, in the same work, *L. casei* ATCC 334 was reported to use l-fucose, which is contradictory to the fact that no *fuc* genes are present in its genome (60). In addition, *L. casei* ATCC 334 was also shown to metabolize 6′FN and N2F *N*-glycan (25), which, according to our results with *L. casei* BL23, can be possible only if the repressor *alfR2* is inactivated.

10.1128/mBio.02804-19.3 FIG S3 Maximum likelihood phylogenetic trees of AlfC (A) and AlfH (B) protein sequences. GenBank accession numbers are indicated in parentheses. Support values higher than 750 for the bootstrap analysis are indicated. The blue bracket indicates the cluster containing the corresponding Lactobacillus casei sequence. Download FIG S3, TIF file, 0.8 MB. Copyright © 2020 Becerra et al. 2020 Becerra et al. This content is distributed under the terms of the Creative Commons Attribution 4.0 International license.

10.1128/mBio.02804-19.4 FIG S4 Maximum likelihood phylogenetic trees of AsnA2 protein sequences. GenBank accession numbers are indicated in parentheses. Support values higher than 750 for the bootstrap analysis are indicated. The blue bracket indicates the cluster containing the corresponding Lactobacillus casei sequence. Download FIG S4, TIF file, 0.7 MB. Copyright © 2020 Becerra et al. 2020 Becerra et al. This content is distributed under the terms of the Creative Commons Attribution 4.0 International license.

10.1128/mBio.02804-19.5 FIG S5 Maximum likelihood phylogenetic trees of AsdA protein sequences. GenBank accession numbers are indicated in parentheses. Support values higher than 750 for the bootstrap analysis are indicated. The blue bracket indicates the cluster containing the corresponding Lactobacillus casei sequence. Download FIG S5, TIF file, 1.0 MB. Copyright © 2020 Becerra et al. 2020 Becerra et al. This content is distributed under the terms of the Creative Commons Attribution 4.0 International license.

10.1128/mBio.02804-19.6 FIG S6 Maximum likelihood phylogenetic trees of PepV protein sequences. GenBank accession numbers are indicated in parentheses. Support values higher than 750 for the bootstrap analysis are indicated. The blue bracket indicates the cluster containing the corresponding Lactobacillus casei sequence. Download FIG S6, TIF file, 1.0 MB. Copyright © 2020 Becerra et al. 2020 Becerra et al. This content is distributed under the terms of the Creative Commons Attribution 4.0 International license.

In bifidobacteria, AsnA2 homologues are present only in Bifidobacterium actinocoloniiforme, Bifidobacterium asteroides, and Bifidobacterium xylocopae (Fig. S4), and as for their AlfC and AlfH counterparts, they are most closely related to *Lactobacillus* sequences. The limited presence in bifidobacteria and the phylogenetic clustering of the three bifidobacterial genes within *Lactobacillus* sequences suggest that they were transferred from *Lactobacillus* to *Bifidobacterium*. Genes encoding PepV homologues are absent in bifidobacteria, whereas *asdA* genes have been detected only in Bifidobacterium magnum and Bifidobacterium gallicum. The AsdA bifidobacterial sequences constitute a well-supported cluster with sequences from the *Bacteroidetes* and are not closely related to their *Lactobacillus* counterparts (Fig. S5), indicating that they had a different evolutionary origin. Interestingly, the closest relatives of *L. casei pepV* are genetically linked to *asnA2* homologues (Fig. 7). This observation suggests a functional link between PepV and AsnA2.

Glycosylasparaginases (EC 3.5.1.26) are essential for the removal of the sugar moiety from the Asn in the GlcNAc-Asn structures derived from *N*-glycoproteins in humans (29, 61). These enzymes cleave the β-aspartylglucosamine linkage, and they require both a free α-amino and α-carboxy group on the Asn substrate (29). In lactobacilli, an *asnA2* homologue had been previously identified in Lactobacillus sakei as a gene induced during meat (sausage) fermentation, and a knockout mutant resulted in reduced growth on meat, but its activity was not ascertained (62). In contrast to the *Elizabethkingia* glycosylasparaginase, AsnA2 from *L. casei* characterized here lacks a signal peptide, strongly suggesting that it is an intracellular enzyme. As described above for glycosylasparaginases, AsnA2 suffers a self-processing proteolytic process to render an active enzyme (Fig. S2). The mature AsnA2 enzyme did not show activity on glycosylated proteins (Fig. S2D), but it showed *in vitro* activity on 6′-fucosylated and nonfucosylated GlcNAc-Asn. Curiously, the presence of the linked l-fucose blocks the hydrolysis of 6′FN-Asn by the human glycosylasparaginase (29). However, for the related PNGases, it has been demonstrated that the size of the carbohydrate moiety in the substrate has little effect on their activity (63). Therefore, AsnA2 would exhibit intermediate characteristics between both types of enzymes: α-1,6-linked l-fucose does not block hydrolysis, and the enzyme does not require a glycosylated peptide as the substrate.

We previously characterized the α-l-fucosidase AlfC from *L. casei* (45) and demonstrated that it displays high regiospecific transglycosylation activity that produces 6′FN disaccharide (47). This enzyme constitutes the only characterized bacterial α-l-fucosidase acting on α-1,6 linkages in core fucosylation structures (core fucosidase), and it was recently employed as a tool for IgG glycoengineering to obtain defucosylated immunoglobulins with enhanced antibody cell-mediated toxicity (51). Core fucosylation of *N*-glycopeptides and *N*-glycoproteins has also been attained using *L. casei* AlfC mutant enzymes (64). In addition to GlcNAc, we show here that AlfC is able to use GlcNAc-Asn, ChbNAc, galactose, and glucose as acceptor substrates in transfucosylation reactions. All the synthesized compounds (6′FN-Asn, N2F *N*-glycan, 6′FucGal, and 6′FucGlc) have exclusively α-1,6-fucosidic bonds, confirming the high linkage specificity of AlfC. However, the recognition of different acceptors indicates a relaxed substrate specificity.

Wild-type *L. casei* did not metabolize the 6′-fucosyl-glycans 6′FN, N2F *N*-glycan, 6′FucGal, and 6′FucGlc, probably due, as shown for 6′FN, to their inability to induce the *alf-2* operon. This was confirmed by inactivating *alfR2*, which resulted in the constitutive expression of *alf-2* genes and the subsequent catabolism of these oligosaccharides. Although *alf-2* operon induction required the complete glycoamino acid 6′FN-Asn, the coexistence of this and other 6′-fucosyl-glycans in environments such as the gastrointestinal tract might allow their simultaneous utilization. Complex networks of cross-feeding between bacteria exist in the gastrointestinal niche, and these substrates are probably released into the gut by the concerted action of different microbial enzymes on glycoproteins and other glycocomplexes (21, 23, 24, 62). The results presented here describe the first catabolic route for the utilization of 6′-fucosyl-related compounds in bacteria. They support previous works that assign to *N*-glycoproteins a role in nourishing beneficial bacteria in the gut. In addition, they show that commensals and pathogens share related mechanisms to take advantage of host molecules.

## MATERIALS AND METHODS

### Transfucosylation reactions.

The AlfC α-l-fucosidase was expressed and purified as previously described (45). The transfucosylation activity of 6×His-AlfC was also analyzed as previously indicated (47), with some modifications. The reaction mixtures (1 ml) contained 100 mM Tris-HCl buffer (pH 7.0); 50 mM *p*-nitrophenyl-α-l-fucopyranoside (*p*NP-fuc) as the donor; and GlcNAc-Asn (100 mM), *N*,*N′*-diacetylchitobiose (150 mM), galactose (150 mM), or glucose (150 mM) as the acceptor. The mixtures were heated at 98°C to solubilize *p*NP-fuc and then cooled to 42°C. Reactions were started by adding 800 U/ml AlfC, and after 20 min (reaction with GlcNAc-Asn as an acceptor), 15 min (reaction with galactose as an acceptor), and 10 min (reactions with *N*,*N′*-diacetylchitobiose or glucose as an acceptor), they were heated at 98°C for 3 min to stop the reaction. The maximum yields obtained for 6*′*FN-Asn, N2F *N*-glycan, 6*′*FucGal, and 6*′*FucGlc were 3.6 g/liter, 1.8 g/liter, 1.3 g/liter, and 3.3 g/liter, respectively. One-dimensional (1D) ^1^H and two-dimensional (2D) ^1^H and ^13^C heteronuclear single quantum coherence (HSQC) and 2D heteronuclear multiple-bond correlation (HMBC) NMR analyses demonstrated the exclusive formation of an α1,6-glycosidic linkage between the sugar monomers.

### Analytical and semipreparative HPLC analyses.

Transfucosylation reaction products were purified by high-performance liquid chromatography (HPLC) using a preparative Rezex RCM-monosaccharide column (Phenomenex) as previously described (65). Appropriate fractions were pooled, concentrated, and analyzed by using an analytical Rezex RSO-oligosaccharide column (Phenomenex) in the case of 6*′*FN-Asn synthesis and an analytical Rezex RCM-monosaccharide column (Phenomenex) in the case of N2F *N*-glycan, 6*′*FucGal, and 6*′*FucGlc synthesis. The synthesized compounds 6*′*FN-Asn, N2F *N*-glycan, 6*′*FucGal, and 6*′*FucGlc were subjected to complete hydrolysis with the α-l-fucosidase AlfC, and the released l-fucose was measured in order to determine their concentrations.

To determine the glycoamino acids and carbohydrates present in the supernatants from the *Lactobacillus* strain cultures, the bacterial cells were removed by centrifugation, and the cultures were analyzed with an ICS3000 chromatographic system (Dionex) using a CarboPac PA100 column with pulsed amperometric detection. A gradient of 10 mM to 100 mM NaOH was used for 16 min at a flow rate of 1 ml/min.

### Nuclear magnetic resonance spectroscopy.

Samples for nuclear magnetic resonance (NMR) spectroscopy were prepared as previously described (66). NMR spectra were also recorded as previously indicated (66), with some modifications. ^1^H-^13^C HSQC data were acquired with 360 transients over a spectral width of 10 ppm (for ^1^H) and 160 to 220 ppm (for ^13^C) and 128 points in the indirect dimension. Total correlation spectroscopy (TOCSY) data were acquired with 64 transients over a spectral width of 10 ppm in both dimensions and 128 points in the indirect dimension. NMR spectra were processed using the program MesReNoeva 8.1 (Mestrelab Research SL).

### Bacterial strains and culture conditions.

*Lactobacillus* strains (Table 1) were grown at 37°C under static conditions in MRS medium (Difco). Escherichia coli was used as a cloning host, and it was routinely grown in Luria-Bertani medium (Oxoid) with shaking at 37°C. The corresponding solid media were prepared by adding 1.8% agar. *L. casei* growth assays with different carbon sources were carried out in MRS basal medium as previously described (41). 6′FN-Asn, 6′FucGal, 6′FucGlc, 6′FN, GlcNAc-Asn, GlcNAc, or glucose was added to MRS basal medium at a concentration of 4 mM, and N2F *N*-glycan was added at 2 mM. Bacterial growth was determined in microtiter plates with a POLARstar Omega plate reader (BMG Labtech). At least three independent biological replicates for each growth curve were obtained, and a representative growth curve is shown. For each biological replicate comparing the wild type and a mutant strain, the same batch of MRS basal medium was used. The concentrations of glycoamino acids and carbohydrates in the culture supernatants at the end of fermentation were determined by HPLC as described above.

**TABLE 1:** Strains and plasmids used in this study^*a*^

Strain or plasmid	Relevant genotype or characteristic(s)	Source or reference
Strains		
*Lactobacillus casei*		
BL23	Wild type	CECT 5275
BL372	BL23 *alfH*::pRV300 Erm^r^	44
BL392	BL23 *sugK*::pRV300 Erm^r^	41
BL405	BL23 *alfR2* (466-bp internal deletion at *alfR2*)	This work
BL406	BL23 *alfR2 alfC* (466-bp internal deletion at *alfR2* and *alfC*::pRV300 Erm^r^)	This work
BL407	BL23 *alfR2 alfH* (466-bp internal deletion at *alfR2* and frameshift in *alfH* at the BclI site)	This work
BL415	BL23 *alfC*::pRV300 Erm^r^	This work
BL416	BL23 *asdA* (frameshift at the HindIII site)	This work
BL417	BL23 *pepV* (frameshift at the NcoI site)	This work
BL418	BL23 *asnA2* (frameshift at the BclI site)	This work
*Escherichia coli*		
DH10B	F^−^ *endA1 recA1 galE15 galK16 nupG rpsL* Δ*lacX74* ϕ80*lacZ*ΔM15 *araD139* Δ(*ara leu*)*7697* *mcrA* Δ(*mrr*-*hsdRMS-mcrBC*) λ^−^	Invitrogen
GM119	F^−^ *supE44 lacY1 galK2 galT22 metB1 dcm-6 dam-3 tsx-78* λ^−^	ATCC 53339
BE50	BL21(DE3) containing pREPGroES/GroEL	80
PE149	DH10B containing pQEalfC	45
PE173	BE50 containing pQEasnA2	This work
PE174	BL21(DE3)/pLys containing pETasdA	This work
PE176	BE50 containing pQEsugK	This work

Plasmids		
pRV300	Suicide vector carrying Erm^r^ from pAMβ1	68
pRValfR2	pRV300 with a fragment carrying a 466-bp deletion at the *alfR2* coding region	This work
pRVasdA	pRV300 with a frameshift at the HindIII site in the *asdA* fragment	This work
pRVpepV	pRV300 with a frameshift at the NcoI site in the *pepV* fragment	This work
pRVasnA	pRV300 with a frameshift at the BclI site in the *ansA* fragment	This work
pRValfC	pRV300 with a 669-bp *alfC* fragment	This work
pRValfH	pRV300 with a frameshift at the BclI site in the *alfH* fragment	This work
pQE80	*E. coli* expression vector; Amp^r^	Qiagen
pET-28a(+)	*E. coli* expression vector; Kan^r^	Novagen
pQEasnA2	pQE80 containing the *asnA2* coding region	This work
pETasdA	pET-28a(+) containing the *asdA* coding region	This work
pQEsugK	pQE80 containing the *sugK* coding region	This work

a CECT, Colección Española de Cultivos Tipo; Erm^r^, erythromycin resistant; Amp^r^, ampicillin resistant; Kan^r^, kanamycin resistant.

E. coli strains were transformed by electroporation with a Gene Pulser apparatus (Bio-Rad), as recommended by the manufacturer. E. coli DH10B transformants were selected with ampicillin (100 μg/ml), and E. coli BE50 transformants were selected with ampicillin (100  μg/ml) and kanamycin (50 μg/ml). *L. casei* strains were transformed as described previously (67). *L. casei* transformants were selected with erythromycin (5  μg/ml).

### Construction of *L. casei* mutants in *alf-2* genes.

*L. casei* BL23 chromosomal DNA was isolated as previously described (67) and used as the template for PCRs, which were performed with the Expand high-fidelity PCR system (Roche). The *alfR2* gene was amplified by PCR using the primer pair AspDecaFor/XhoPeptVRev2 (see Table S3 in the supplemental material). The resulting 1,289-bp fragment was ligated into pRV300 (68) digested previously with EcoRV/KpnI and treated with the Klenow fragment of DNA polymerase I. The obtained plasmid was digested with SalI, ligated, and transformed. A clone with a 466-bp deletion in *alfR2* (pRValfR2) was selected. DNA fragments containing parts of *asdA*, *pepV*, and *asnA2* were obtained by PCR using the oligonucleotides pairs AsdFor/AsdRev, PepVFor/PepVRev, and AsnFor/AsnRev, respectively (Table S3), and cloned into pRV300 digested with EcoRV. The resulting plasmids, pRVasdA, pRVpepV, and pRVasnA, were cleaved at the unique HindIII, NcoI, and BclI restriction sites present in the *asdA*, *pepV*, and *asnA2* coding regions, respectively, to introduce frameshifts into their corresponding coding sequences (Table 1). The three digested plasmids were then treated with Klenow fragment, ligated, and transformed into E. coli DH10B. The resulting plasmids were transformed into *L. casei* BL23, and frameshifts were introduced into the corresponding genes by a double-recombination strategy (41) (Table 1). To construct an *alfC* mutant, an internal DNA fragment of *alfC* was obtained by PCR using the oligonucleotides AlfCFor and AlfCRev. The PCR product was cloned into pRV300 digested with EcoRV. The resulting plasmid, pRValfC, was used to transform *L. casei* BL23, and single-crossover integrants were selected by resistance to erythromycin and confirmed by PCR analysis and DNA sequencing. One mutant was selected and named BL415. The same procedure was used to inactivate *alfC* in the mutant strain BL405 (*alfR2*), obtaining the *alfR2 alfC* double mutant (strain BL406). To construct the *alfR2 alfH* double mutant (strain BL407), the plasmid pRValfH was generated (Table 1). A DNA fragment containing part of *alfH* was obtained with the oligonucleotide pair FucPerFor/FucPerRev and cloned into pRV300 digested with EcoRV. The resulting plasmid, pRValfH, was cleaved at the unique BclI restriction site present in the *alfH* coding region, treated with Klenow fragment, ligated, and transformed. A construct in which a frameshift was introduced at the BclI site in *alfH* (pRValfH) was selected. pRValfH was transformed into the mutant strain BL405 (*alfR2*). A clone having a second recombination event was selected to obtain the *alfR2 alfH* double mutant (strain BL407).

10.1128/mBio.02804-19.9 TABLE S3 Primers used in this study. Download Table S3, DOCX file, 0.01 MB. Copyright © 2020 Becerra et al. 2020 Becerra et al. This content is distributed under the terms of the Creative Commons Attribution 4.0 International license.

### Sequence analysis.

DNA sequencing was carried out by the Central Service of Research Support of the University of Valencia (Spain). M13 universal and reverse primers or custom primers hybridizing within the appropriate DNA fragments were used for sequencing. Sequence analyses were carried out with DNAMAN 4.03 for Windows (Lynnon BioSoft), and sequence similarities were analyzed with the BLAST program (69). Genomic context analysis was performed at the Microbial Genome Database for Comparative Analysis (MBGD) (http://mbgd.genome.ad.jp/) (70).

### Reverse transcription-quantitative PCR analysis.

Total RNA was isolated from *L. casei* strains BL23 (WT) and BL405 (*alfR2*) grown in MRS basal medium supplemented with 4 mM different glycans (6′FN-Asn, 6′FN, GlcNAc, or glucose), as previously described (41). The isolated RNA was digested with DNase I and retrotranscribed using the Maxima first-strand cDNA synthesis kit (Fermentas) (41). Reverse transcription-quantitative PCR (RT-qPCR) was performed for each cDNA sample in triplicate using the LightCycler 480 system (Roche), LC fast-start DNA master SYBR green I (Roche), and the primers pairs q29280for/q29280rev (*sugK*), q29290for/q29290rev (*asnA2*), q29300for/q29300rev (*pepV*), q29310for/q29310rev (*alfR2*), q29320for/q29320rev (*asdA*), q29330for/q29330rev (*alfH*), and q29340for/q29340rev (*alfC*) (Table S3). The reaction mixtures and cycling conditions were previously described (41). The *pyrG*, *lepA*, and *ileS* genes were chosen as reference genes (71). Relative expression values were calculated using the software tool REST (relative expression software tool) (72). Linearity and amplification efficiency were determined for each primer pair.

### Expression and purification of AsnA2, AsdA, and SugK.

The coding regions of *asnA2*, *asdA*, and *sugK* were amplified by PCR using the primer pairs AsnHindIIFor/AsnBamHIRev, AsdAHCt-F/AsdAHCt-R, and SugKBamH1For/SugKRev, respectively (Table S3). AsnA2 was cloned into the HindIII-BamHI sites and SugK was cloned into the BamHI-SmaI sites of the pQE80 vector and transformed into E. coli BE50 coexpressing the chaperones *groES* and *groEL*. pETasdA was constructed using the Gibson assembly kit (NEB) with the *asdA* PCR fragment and pET28a(+) digested with NcoI and XhoI. pETasdA was transformed into E. coli BL21(DE3)/pLys. For purification, cells were grown to an OD at 600 nm (OD_600_) of 0.8, 1 mM isopropyl-β-d-thiogalactopyranoside was added, and incubation was continued at 25°C for 10 h. Bacterial cell extracts were loaded onto a HisTrap column (GE Healthcare), and His-tagged protein was purified according to the supplier’s recommendations. The native molecular weight of the AsnA2 protein was estimated by size exclusion chromatography (HiPrep 16/60 Sephacryl S-300 HR column).

### Mass spectrometry analysis.

Protein bands from AsnA2 purification were excised from the gel, trypsinized, and analyzed with a 5800 matrix-assisted laser desorption ionization–tandem time of flight (MALDI-TOF/TOF) system (AB Sciex) at the proteomics service of the University of Valencia. MS and tandem MS (MS/MS) data were analyzed with the Mascot server.

### AsnA2 enzyme activity.

The activity of the purified AsnA2 enzyme was assayed at 37°C with GlcNAc-Asn and 6′FN-Asn as the substrates. The released GlcNAc and 6′FN, respectively, were quantified by analyzing the reaction mixtures by ionic chromatography (Dionex) as described above. Reactions using mixtures (20 μl) containing 5 mM the substrate in 100 mM Tris-HCl buffer (pH 7.0) were initiated by adding 0.2 μg of the enzyme. Protein deglycosylation assays were performed with reaction mixtures (15 μl) containing 3.75 μg of ovalbumin or lactoferrin in 100 mM Tris-HCl buffer (pH 7.0). The reactions were initiated with 0.2  μg of the enzyme, and the mixtures were incubated at 37°C overnight.

### AsdA enzyme activity.

AsdA decarboxylase activity on l-Asp was measured as previously described, with some modifications (73). The resulting l-Ala was detected by using a coupled enzyme assay. Reactions using mixtures (100 μl) containing 100 mM K-acetate buffer (pH 5.5), 0.2 mM pyridoxal 5-phosphate, 1 mM α-ketoglutarate, 0.4 mM NAD^+^, 0.32 U of l-alanine dehydrogenase (Sigma), and 10 μg of AsdA were initiated by the addition of 40 mM l-Asp. Reaction mixtures were incubated for 60 min at 37°C, and the production of NADH was monitored at 340 nm.

### SugK enzyme activity.

SugK phosphorylation activity on different sugars was determined by using a coupled enzyme assay as previously described (74). Reactions using mixtures (100 μl) containing 100 mM Tris-HCl (pH 7.5), 10 mM MgCl_2_, 5 mM ATP, 1 mM phosphoenolpyruvate, 0.2 mM NADH, 10 U of pyruvate kinase, 7 U of lactate dehydrogenase, and various substrates (GlcNAc, Glc, Gal, or GalNAc) at a final concentration of 1 mM were initiated by the addition of 0.75 μg of SugK. Reaction mixtures were incubated for 15 min at 37°C, and NADH formation was monitored at 340 nm.

### Phylogenetic analysis.

Sequences of *alfC*, *alfH*, *asnA2*, *asdA*, and *pepV* homologues were retrieved from the microbial genome repository at the NCBI by BLAST (75) using their corresponding *L. casei* BL23 protein sequences as query sequences. For *asnA2*, only sequences from the *Lactobacillaceae* and *Bifidobacteriaceae* were included. Domain analysis was performed using the tools implemented at the NCBI BLAST site (https://blast.ncbi.nlm.nih.gov/Blast.cgi). The resulting data sets were aligned with M-Coffee (76) at the T-Coffee server (http://tcoffee.crg.cat), with default settings. Positions of uncertain homology and gaps were removed using GBLOCKS (77) at the Gblocks server (http://molevol.cmima.csic.es/castresana/Gblocks_server.html), allowing smaller final blocks and less strict flanking positions. Redundant sequences were removed by using the EMBOSS suite Skipredundant tool (78) with a percent sequence identity redundancy threshold of 98%. The best-fit models of amino acid substitution and maximum likelihood trees were obtained using PhyML version 3.0 (79) at the PhyML server (http://www.atgc-montpellier.fr/phyml). Bootstrap support values were obtained from 1,000 pseudorandom replicates.

### Statistical analysis.

Student’s *t* test was performed using Statgraphics Plus version 2.1 (Statistical Graphics Corp., USA), and it was used to detect statistically significant differences between the final OD values reached by *L. casei* BL405 (*alfR2*) cultures and the mutant strains BL406 (*alfR2 alfC*) and BL407 (*alfR2 alfH*). Statistical significance was accepted at a *P* value of <0.05.
