# Supplementary figures and images for: Unique Microbial Catabolic Pathway for the Human Core : N-Glycan Constituent Fucosyl-α-1,6-N-Acetylglucosamine-Asparagine
Source: mBio. 2020 Jan 14;11(1):e02804-19. doi: 10.1128/mBio.02804-19 (PMC6960285; doi:10.1128/mBio.02804-19)

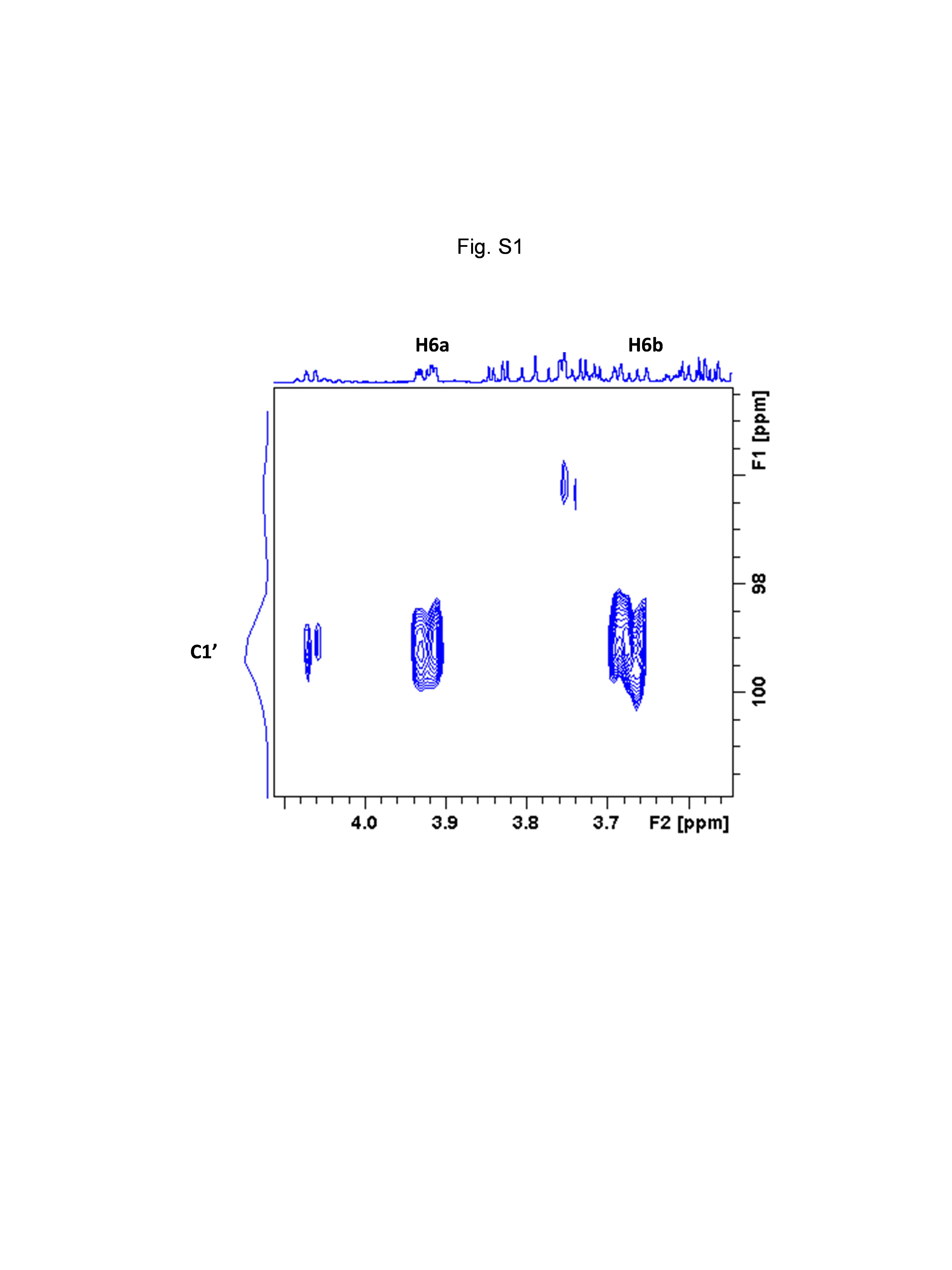

Supplement: FIG S1 [file mBio.02804-19-sf001.tif]

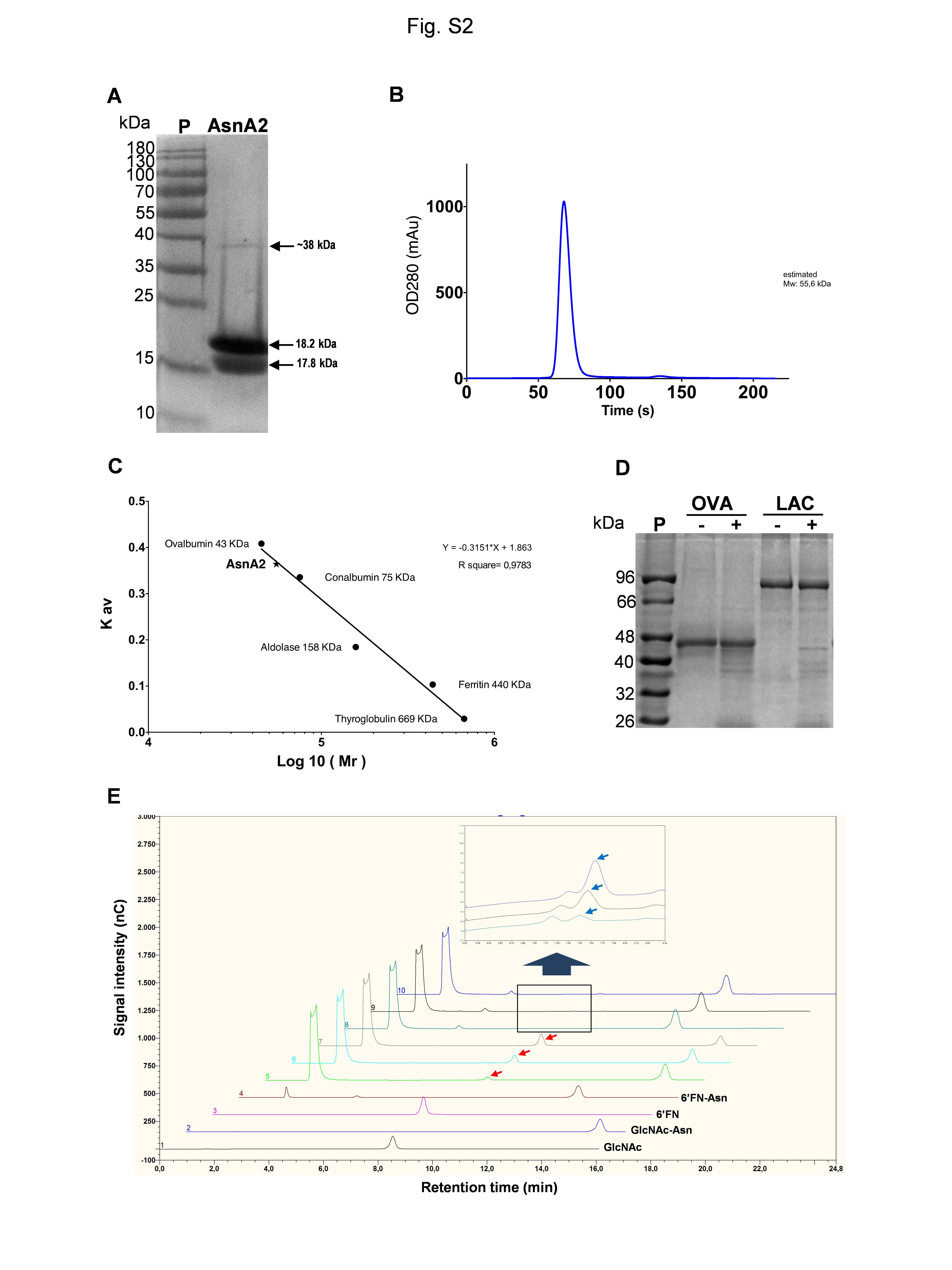

Supplement: FIG S2 [file mBio.02804-19-sf002.tif]

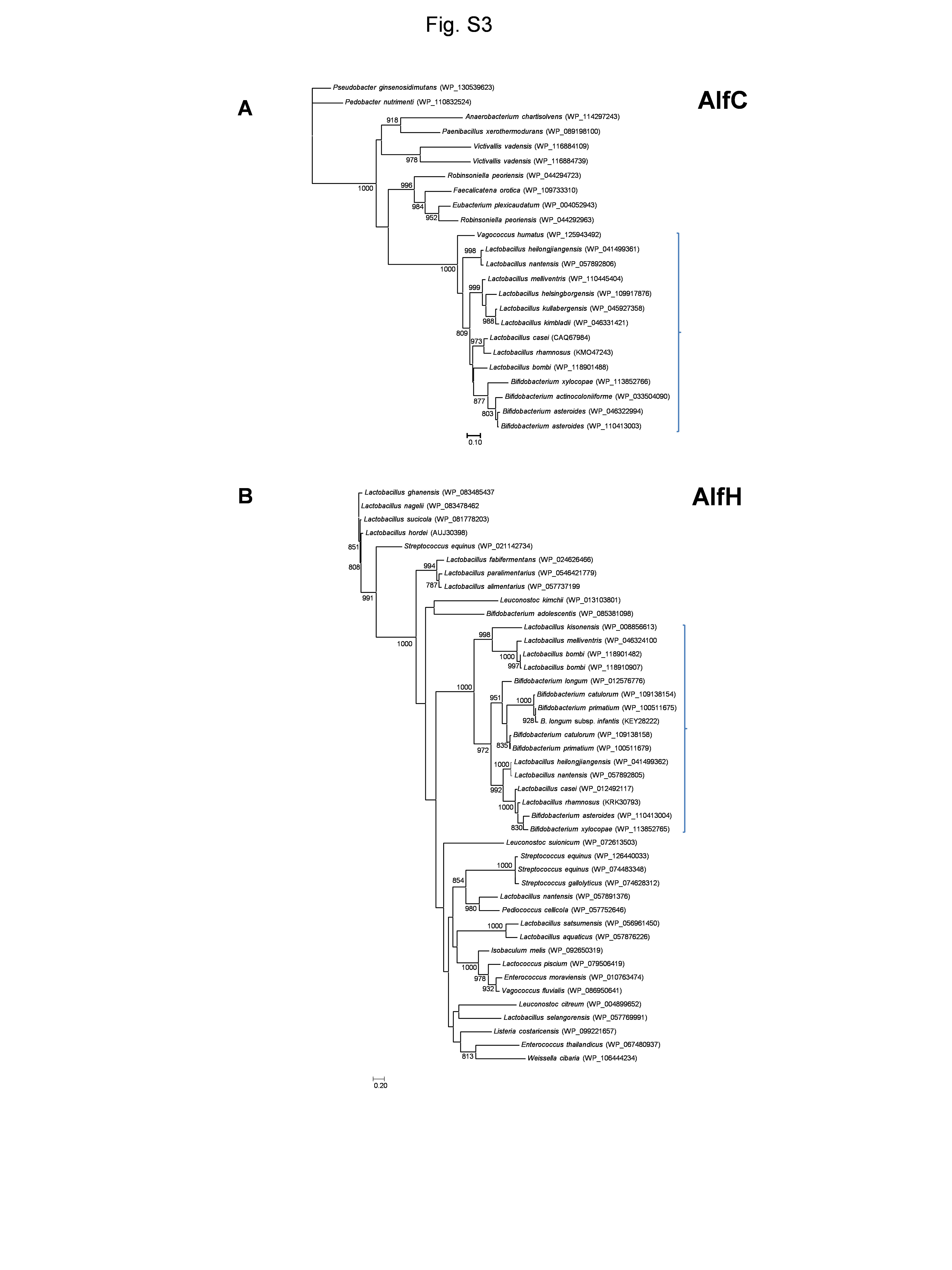

Supplement: FIG S3 [file mBio.02804-19-sf003.tif]

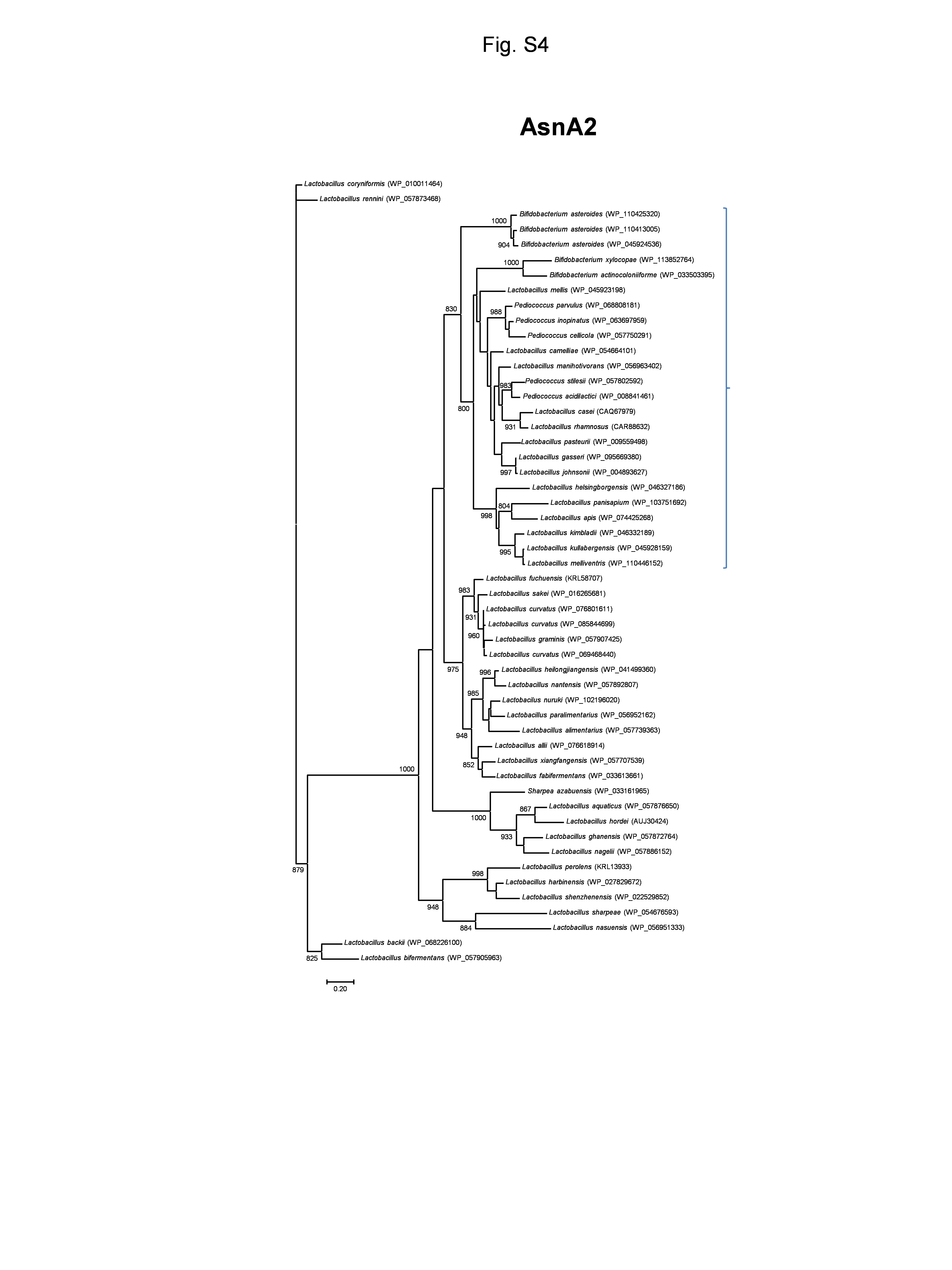

Supplement: FIG S4 [file mBio.02804-19-sf004.tif]

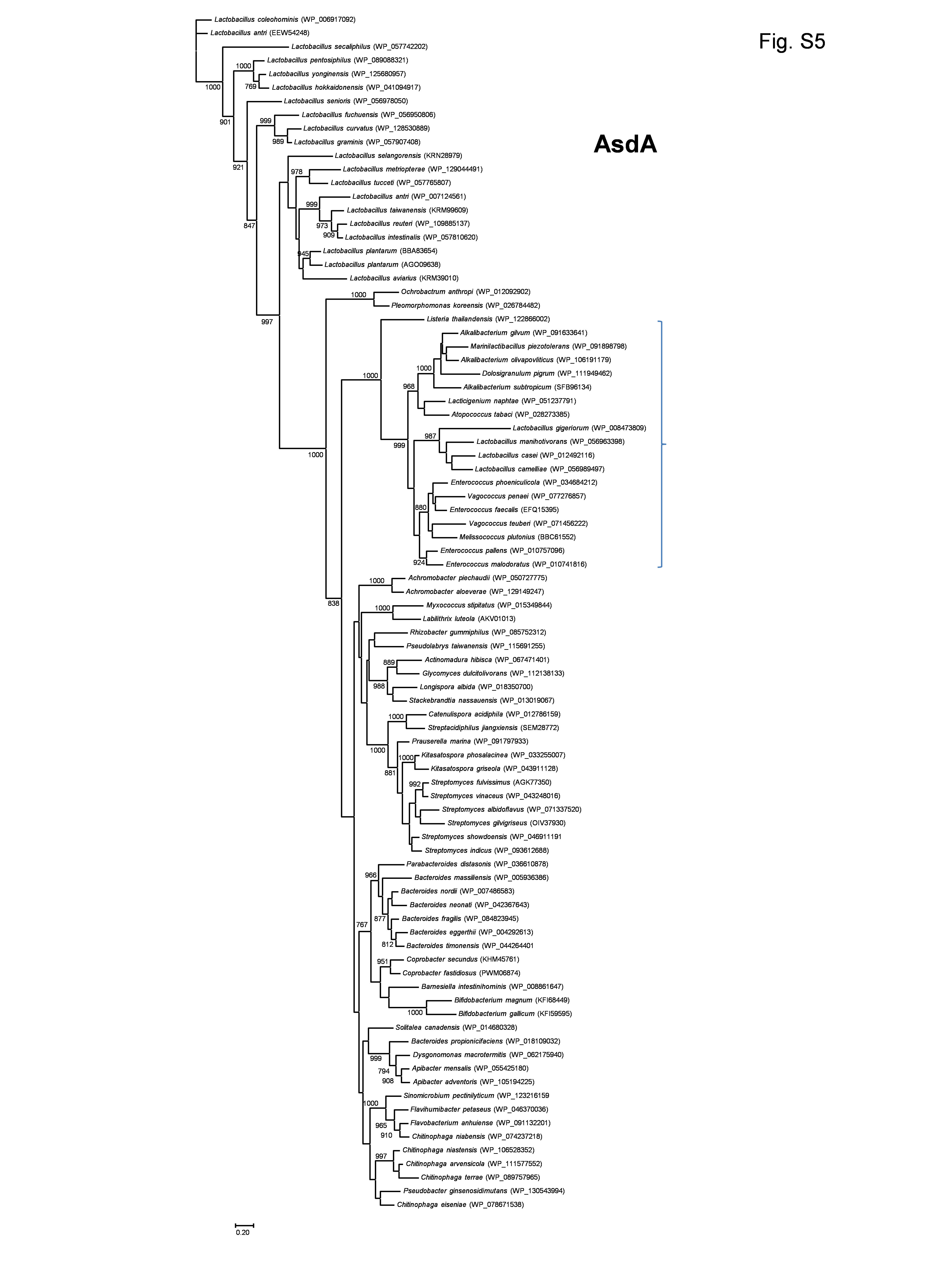

Supplement: FIG S5 [file mBio.02804-19-sf005.tif]

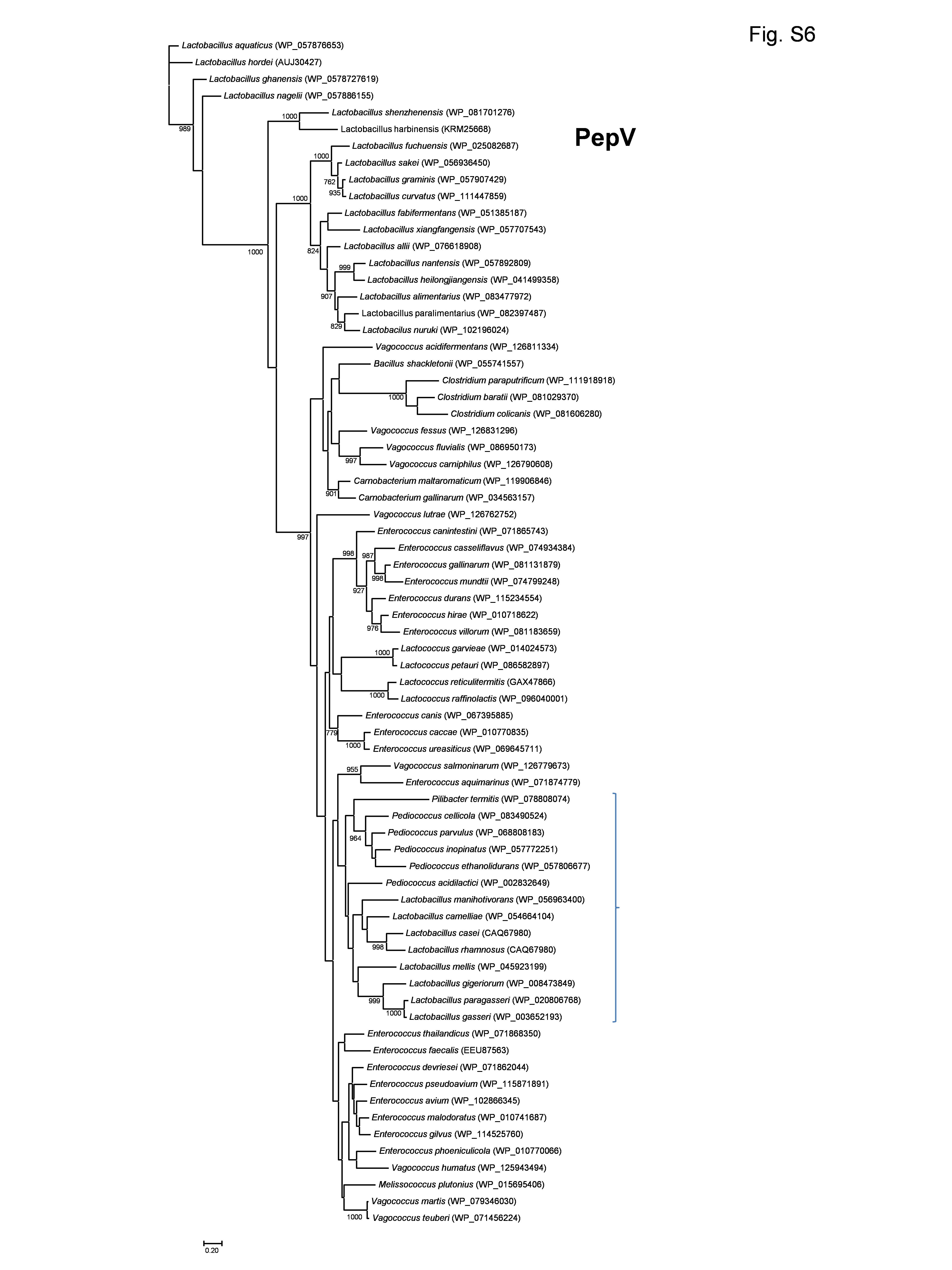

Supplement: FIG S6 [file mBio.02804-19-sf006.tif]
