# Supplementary material for: Unique Microbial Catabolic Pathway for the Human Core : N-Glycan Constituent Fucosyl-α-1,6-N-Acetylglucosamine-Asparagine
Source: mBio. 2020 Jan 14;11(1):e02804-19. doi: 10.1128/mBio.02804-19 (PMC6960285; doi:10.1128/mBio.02804-19)
Supplement: TABLE S1 [file mBio.02804-19-st001.docx]

**Table S1.** ^1^H and ^13^C assignment of compounds fucosyl-α-1,6-*N*-acetylglucosamine-asparagine, fucosyl-α-1,6-*N*,N’-diacetylchitobiose, fucosyl-α-1,6-galactose and fucosyl-α-1,6-glucose carried out at 27 °C and 600 MHz in D_2_O. The spectrum was referenced to the water signal at 4.7 ppm.

| atom | group | δ^1H^ (ppm) | multiplicity | J (Hz) | δ^13C^ (ppm) |
| --- | --- | --- | --- | --- | --- |
| **fucosyl-α-1,6-*N*-acetylglucosamine-asparagine** | | | | | |
| 1 | CH | 5.04 | d | 10 (H2) | 74.4 |
| 2 | CH | 3.79 | t | 10 (H1, H3) | 54.9 |
| 3 | CH | 3.56 | m | - | 69.8 |
| 4 | CH | 3.61 | m | - | 72.8 |
| 5 | CH | 3.49 | m | - | 64.2 |
| 6 | CH_2_ | 3.92/3.68 | m | - | 62.4 |
| 7 | NHCO | - | - | - | 172.5 |
| 8 | CH_2_ | 2.85 | ddd | 31/17/7 | 102.2 |
| 9 | CH | 3.92 | m | - | 121.8 |
| 10 | COOH | - | - | - | 173.4 |
| 11 | NHCO | - | - | - | 86.6 |
| 12 | CH_3_ | 1.97 | s | - | 174.8 |
| 1’ | CH | 4.85 | d | 4 (H2’) | 99.7 |
| 2’ | CH | 3.72 | m | - | 62.5 |
| 3’ | CH | 3.76 | m | - | 66.9 |
| 4’ | CH | 3.84 | m | - | 64.2 |
| 5’ | CH | 3.75 | m | - | 65.7 |
| 6’ | CH_3_ | 1.16 | d | 7(H5’) | 78.5 |
| **fucosyl-α-1,6-*N*,N’-diacetylchitobiose** | | | | | |
| 1 | CH | 5.09 | d | 3 (H2) | 90.4 |
| 2 | CH | 3.81 | m | - | 53.2 |
| 3 | CH | 3.79 | m | - | 69.5 |
| 4 | CH | 3.83 | m | - | 69.5 |
| 5 | CH | 3.50 | m | - | 80.7 |
| 6 | CH2 | 3.56/3.71 | m | - | 59.8 |
| 7 | NHCO | - | - | - | 174.3 |
| 8 | CH3 | 1.98 | m | - | 22.04 |
| 1’ | CH | 4.51 | m | - | 101.6 |
| 2’ | CH | 3.72 | m | - | 71.6 |
| 3’ | CH | 3.49 | m | - | 73.4 |
| 4’ | CH | 3.61 | m | - | 59.5 |
| 5’ | CH | 3.46 | m | - | 69.7 |
| 6’ | CH2 | 3.94/3.67 | m | - | 67.2 |
| 7’ | NHCO | - | - | - | 174.3 |
| 8’ | CH3 | 1.98 | m | - | 22.2 |
| 1’’ | CH | 4.82 | t | 4 (H2’’, H3’’) | 99.2 |
| 2’’ | CH | 3.67 | m | - | 68.4 |
| 3’’ | CH | 3.81 | m | - | 69.0? |
| 4’’ | CH | 3.73 | m | - | 56.7 |
| 5’’ | CH | 4.00 | q | 6 (H6’’) | 66.7 |
| 6’’ | CH3 | 1.14 | d | 5 (H5’’) | 15.1 |
| **fucosyl-α-1,6-galactose** | | | | | |
| 1a  1b | CH  CH | 5.21  4.52 | d | 4 (H2a)  8 (H2b) | 92.2  96.4 |
| 2a  2b | CH  CH | 3.76  3.45 | m  dd | -  8 (H1b), 10 (H3b) | 68.0  71.8 |
| 3a  3b | CH  CH | 3.80  3.59 | m  dd | -  4 (H4b), 10 (H2b) | 69.4  72.7 |
| 4a  4b | CH  CH | 3.94  3.88 | dd  dd | 3(H5a), 1(3a)  4 (H3b), 1 (H5b) | 69.4  68.8 |
| 5a  5b | CH  CH | 4.21  3.85 | dd  dd | 8 (H6a), 3 (H4a) | 69.2  73.5 |
| 6a  6b | CH_2_  CH_2_ | 3.77/3.67  3.79/3.66 | m  dd | 8 (H5), 3 (H6) | 67.3  67.3 |
| 1’ | CH | 4.87 | t | 4 | 99.02 |
| 2’ | CH | 3.73 | m | - | 68.0 |
| 3’ | CH | 3.75 | m | - | 71.8 |
| 4’ | CH | 3.81 | m | - | 69.4 |
| 5’ | CH | 4.04 | q | 7(H6’) | 66.7 |
| 6’ | CH_3_ | 1.16 | d | 7(H5’) | 15.1 |
| **fucosyl-α-1,6-glucose** | | | | | |
| 1a  1b | CH  CH | 5.14  4.56 | d  d | 4 (H2a)  8 | 92.0  95.9 |
| 2a  2b | CH  CH | 3.46  3.17 | dd  t | 10 (H3a), 4 (H1a)  8 (H1b, 3b) | 71.3  73.9 |
| 3a  3b | CH  CH | 3.63  3.41 | m  t | -  9 (H2b, H4b) | 72.5  75.1 |
| 4a  4b | CH  CH | 3.89  3.38 | m  m | -  - | 70.6  75.5 |
| 5a  5b | CH  CH | 3.36  3.53 | t  m | 9 (H4a, H6a)  - | 69.5  74.8 |
| 6a  6b | CH_2_  CH_2_ | 3.84/3.68  3.89/3.62 | m  m | -  - | 67.6  67.2 |
| 1’ | CH | 4.84/4.83 | d | 4(H2’) | 99.1 |
| 2’ | CH | 3.70 | m | - | 68.1 |
| 3’ | CH | 3.81 | m | - | 69.4 |
| 4’ | CH | 3.72 | m | - | 71.6 |
| 5’ | CH | 4.03 | s | 7 (H6’, H4’) | 66.7 |
| 6’ | CH_3_ | 1.14 | d | 7 (H6’) | 15.1 |
