# Supplementary material for: Unique Microbial Catabolic Pathway for the Human Core : N-Glycan Constituent Fucosyl-α-1,6-N-Acetylglucosamine-Asparagine
Source: mBio. 2020 Jan 14;11(1):e02804-19. doi: 10.1128/mBio.02804-19 (PMC6960285; doi:10.1128/mBio.02804-19)
Supplement: TABLE S2 [file mBio.02804-19-st002.docx]

**Table S2**. Final O.D. values (means ± standard deviations) reached by *Lactobacillus casei* mutant strains cultured on 6’fucosyl glycans.

| **Strain** | **6’fucosyl glycans** | **O.D. _550_** | ***P* value*** |
| --- | --- | --- | --- |
| BL405 (*alfR2*) | 6’FN | 0.628 ± 0.013 |  |
|  | 6’FucGlc | 0.696 ± 0.100 |  |
|  | 6’FucGal | 0.675 ± 0.057 |  |
|  | N2F *N*-glycan | 0.815 ± 0.120 |  |
|  |  |  |  |
| BL406 (*alfR2 alfC*) | 6’FN | 0.419 ± 0.016 | 0.001 |
|  | 6’FucGlc | 0.438 ± 0.014 | 0.022 |
|  | 6’FucGal | 0.473 ± 0.014 | 0.002 |
|  | N2F *N*-glycan | 0.529 ± 0.062 | 0.03 |
|  |  |  |  |
| BL407 (*alfR2 alfH*) | 6’FN | 0.495 ± 0.037 | 0.022 |
|  | 6’FucGlc | 0.491 ± 0.018 | 0.012 |
|  | 6’FucGal | 0.490 ± 0.022 | 0.003 |
|  | N2F *N*-glycan | 0.487 ± 0.006 | 0.052 |

* *p*-value between final O.D. values reached by *L. casei* BL405 (*alfR2*) cultures versus each mutant BL406 (*alfR2 alfC*) and BL407 (*alfR2 alfH*) strains.

6’FN, fucosyl-α-1,6-*N*-acetylglucosamine; 6’FucGlc, fucosyl-α-1,6-glucose; 6’FucGal, fucosyl-α-1,6-galactose; N2F *N*-glycan, fucosyl-α-1,6-*N*,*N*’-diacetylchitobiose.
