# Supplementary material for: Unique Microbial Catabolic Pathway for the Human Core : N-Glycan Constituent Fucosyl-α-1,6-N-Acetylglucosamine-Asparagine
Source: mBio. 2020 Jan 14;11(1):e02804-19. doi: 10.1128/mBio.02804-19 (PMC6960285; doi:10.1128/mBio.02804-19)
Supplement: TABLE S3 [file mBio.02804-19-st003.docx]

**Table S3**. Primers used in this study

| **Name** | **Sequence** |
| --- | --- |
| AspDecaFor | CGATATGGTGCTGTTTTTGC |
| XhoPeptVRev2 | CATTGCTCGAGTGCCATGGCATCATTGACG |
| AsdFor | TGAAAATGAATAAAGGCAGGC |
| AsdRev | CACAAAAGGGAGCGTATTAGC |
| PepVFor | GTTCCGTGATCGCATTGACG |
| PepVRev | ATGGCCAAACTCTTGCATGG |
| AsnFor | CATGATCCATGGCGATTAGG |
| AsnRev | CGCATAAAGAAAATGAGTGG |
| AlfCFo**r** | ATCCGCTGTACTTCGATGC |
| AlfCRev | AAGAATCATT AATGGTGCC |
| FucPerFor | TATGATGTGTATGTACCAAGC |
| FucPerRev | AACAGAACATAGAAGATGCCG |
| K29280For | GTCTCGTTAAAGCTAGTGC |
| K29280Rev | CGGTTTGCATGTTGTAGATC |
| q29280for | TAAGACAACCATGGCGCTGT |
| q29280rev | AAAATGACGGTAATGCGGCG |
| q29290for | CTTGGCATCAGCGATTTCCG |
| q29290rev | AATACGCCCAACTCAACGGT |
| q29300for | AAGTGCCCGACAATTCCGAT |
| q29300rev | CTATGGCTTCAAAACGGCGG |
| q29310for | CGACGGTCATTGGTTCCTCA |
| q29310rev | CAGAAGCGGCCAAAGCATAC |
| q29320for | CAAGTGTCACTGCCAAACGG |
| q29320rev | CTCGCTTGCTAGTCGCCTTA |
| q29330for | TCACCACTTCCATTCCGGTG |
| q29330rev | GGTTAGTGCCGGGTTGATGA |
| q29340for | ACATTGCAACAGCGTGGTTC |
| q29340rev | TCGCCCAGAGAAACGAAGTC |
| AsnHindIIIFor | CCAGAACGAAGGAAGCTTCATTCAATCG |
| AsnBamHIRev | GGAGGGATGGATCCATGAGCAACGAAAC |
| AsdAHCt-F | AAGAAGGAGATATACCATGACCATCTCGATCGATGCACTC |
| AsdAHCt-R | GGTGGTGGTGGTGCTCGAGTTTGGCCTCCGTTTCACATG |
| SugKBamH1For | TCGGTGAAAAGGAGTTAGGATCCCAGTATTTAGCGATTGA |
| SugKRev | TTACTACCCGCGCACCACCAACGGCGCCACC |
